# A NRF2 Regulated and the Immunosuppressive Microenvironment Reversed Nanoplatform for Cholangiocarcinoma Photodynamic‐Gas Therapy

**DOI:** 10.1002/advs.202307143

**Published:** 2024-02-02

**Authors:** Weimin Wang, Yang Gao, Jianjun Xu, Tianhao Zou, Bin Yang, Shaobo Hu, Xiang Cheng, Yun Xia, Qichang Zheng

**Affiliations:** ^1^ Department of Hepatobiliary Surgery Union Hospital Tongji Medical College Huazhong University of Science and Technology Wuhan 430022 China; ^2^ Liver Transplant Center Union Hospital Tongji Medical College Huazhong University of Science and Technology Wuhan 430022 China; ^3^ Department of Digestive Oncology Surgery Cancer Centre Union Hospital Tongji Medical College Huazhong University of Science and Technology Wuhan 430022 China; ^4^ Department of General Surgery Tongji Hospital Tongji Medical College Huazhong University of Science and Technology Wuhan 430030 China

**Keywords:** ferroptosis, gas therapy, NRF2, photodynamic therapy, tumor microenvironment reversal

## Abstract

Photodynamic therapy (PDT) is a minimally invasive and controllable local cancer treatment for cholangiocarcinoma (CCA). However, the efficacy of PDT is hindered by intratumoral hypoxia and the presence of an antioxidant microenvironment. To address these limitations, combining PDT with gas therapy may be a promising strategy to enhance tumor oxygenation. Moreover, the augmentation of oxidative damage induced by PDT and gas therapy can be achieved by inhibiting NRF2, a core regulatory molecule involved in the antioxidant response. In this study, an integrated nanotherapeutic platform called CMArg@Lip, incorporating PDT and gas therapies using ROS‐responsive liposomes encapsulating the photosensitizer Ce6, the NO gas‐generating agent L‐arginine, and the NRF2 inhibitor ML385, is successfully developed. The utilization of CMArg@Lip effectively deals with challenges posed by tumor hypoxia and antioxidant microenvironment, resulting in elevated levels of oxidative damage and subsequent induction of ferroptosis in CCA. Additionally, these findings suggest that CMArg@Lip exhibits notable immunomodulatory effects, including the promotion of immunogenic cell death and facilitation of dendritic cell maturation. Furthermore, it contributes to the anti‐tumor function of cytotoxic T lymphocytes through the downregulation of PD‐L1 expression in tumor cells and the activation of the STING signaling pathway in myeloid‐derived suppressor cells, thereby reprogramming the immunosuppressive microenvironment via various mechanisms.

## Graphical abstract

Photodynamic therapy is a promising minimally invasive treatment for tumors, but limited by tumor hypoxia and antioxidant microenvironment. This study constructs a nanoplatform that combines photodynamic and gas therapy to combat hypoxic microenvironments. Simultaneously blocking NRF2 signaling enhances oxidative damage caused by ROS and RNS, inducing ferroptosis and reshaping the immune microenvironment, effectively activating anti‐tumor immunity.

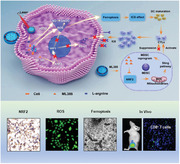

## 1 Introduction

Photodynamic therapy (PDT) is an effective modality for tumor treatment,^[^ ^1^, ^2^, ^3^ ^]^ and its application in the management of malignancies, such as cholangiocarcinoma (CCA),^[^ ^4^, ^5^, ^6^ ^]^ has demonstrated definitive therapeutic benefits. Besides its direct cytotoxicity toward tumor cells, PDT exhibits immunomodulatory properties supported by accumulating evidence.^[^ ^7^, ^8^, ^9^ ^]^ It induces immunogenic cell death (ICD),^[^ ^10^, ^11^ ^]^ thereby facilitating the release of tumor antigens^[^ ^12^ ^]^ and recruitment of cytotoxic T lymphocytes (CTLs),^[^ ^13^ ^]^ consequently enhancing the anti‐tumor immune microenvironment. Nevertheless, several challenges persist in the utilization of PDT. First, its killing efficiency remains suboptimal due to tumor tissue hypoxia^[^ ^14^, ^15^, ^16^ ^]^ and the inherent antioxidant system within cells,^[^ ^17^, ^18^ ^]^ leading to limited reactive oxygen species (ROS) generation or increased scavenging. Second, PDT induces the expression of programmed death‐ligand 1 (PD‐L1) in tumor cells,^[^ ^19^, ^20^ ^]^ promoting the establishment of adaptive immune tolerance. These factors significantly compromise the therapeutic efficacy of PDT.

The nuclear factor erythroid 2‐related factor 2 (NRF2) plays a crucial role in maintaining intracellular redox homeostasis^[^ ^21^, ^22^, ^23^ ^]^ and emerges as a significant obstacle to be surmounted in the context of PDT.^[^ ^24^, ^25^, ^26^ ^]^ Our investigation unveils a heightened expression of NRF2 in CCA, and PDT stimulation further amplifies NRF2 activity, substantially blunting the oxidative damage evoked by PDT. Furthermore, earlier studies have demonstrated the direct involvement of NRF2 as a transcription factor for PD‐L1.^[^ ^27^, ^28^ ^]^ Consequently, the upregulation of NRF2 induced by PDT engenders an elevation in PD‐L1 expression in CCA, detrimentally impeding the T cell‐mediated anti‐tumor immune response. Moreover, it is worth emphasizing the noteworthy observation that myeloid‐derived suppressor cells (MDSCs) represent an immunosuppressive cellular subset within the tumor microenvironment (TME),^[^ ^29^ ^]^ and abrogation of their NRF2 signaling instigates a reprogramming of MDSCs from an immunosuppressive phenotype to one that promotes the efficacy of CTLs.^[^ ^30^, ^31^ ^]^ These findings underscore the promising potential of targeting NRF2 signaling as a valuable therapeutic strategy to enhance PDT efficiency and reverse immune tolerance.

Nitric oxide (NO), a pleiotropic and highly significant gaseous transmitter, has been utilized in gas therapy for the treatment of cancer,^[^ ^32^, ^33^ ^]^ along with its potential as an adjunctive therapy for PDT. At concentrations ranging from 1 µm to 1 mm, NO can directly eliminate cancer cells, while causing minimal adverse effects. Additionally, NO can enhance both gas and photodynamic therapeutic effects by reacting with ROS, resulting in the production of highly reactive peroxynitrite (ONOO^•^) molecules^[^ ^34^ ^]^ with significantly increased antitumor activity. Moreover, recent studies have demonstrated that NO possesses immunomodulatory properties. It can modify the immunosuppressive TME by inducing ICD,^[^ ^35^ ^]^ promoting the polarization of macrophages to M1 phenotype,^[^ ^36^ ^]^ increasing T cell infiltration, and reducing PD‐L1 expression.^[^ ^37^ ^]^ Furthermore, delivery of NO using nanocarriers can improve tumor vascular normalization, effectively ameliorate the hypoxic microenvironment of tumor tissues, and enhance anticancer therapy. Various NO‐delivery agents have been developed for NO‐based gas therapy. Notably, L‐Arginine (Arg), a natural NO donor with excellent biocompatibility,^[^ ^38^ ^]^ can continuously release intratumoral NO in a stimuli‐responsive manner in H_2_O_2_‐rich TME, making it ideal for gas therapy.

**Scheme 1:**
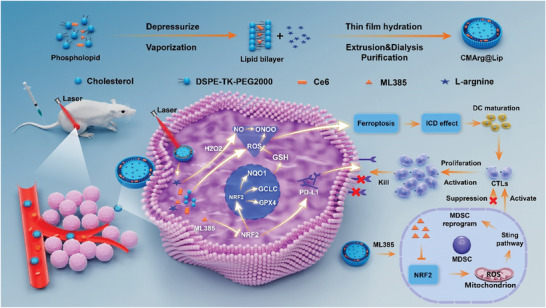
Schematic illustration of the photodynamic‐gas therapy mediated by CMArg@Lip for the reversal of the tumor immunosuppressive microenvironment. Upon accumulation in the tumor region, CMArg@Lip undergoes photostimulation‐induced ROS generation, resulting in the release of the encapsulated drug payload. Furthermore, H_2_O_2_ facilitates the oxidation of Arg, leading to the production of NO which subsequently reacts with ROS generated by Ce6 to form the highly potent ONOO^−^. Additionally, ML385 counteracts the intrinsic antioxidant capacity of cancer cells. The treatment with CMArg@Lip induces ferroptosis in cancer cells, thereby triggering a more enhanced ICD effect and facilitating the down‐regulation of PD‐L1 expression as well as the reprogramming of MDSCs, ultimately resulting in the effective reversal of the tumor immunosuppressive microenvironment.

Hence, it is postulated that the synergistic photodynamic‐gaseous therapeutic effect on CCA can be achieved by combining chlorin e6 (Ce6) and NO donor (Arg) photosensitizers with an NRF2 inhibitor (ML385). In order to address the aforementioned challenges, we have developed a multifunctional nanoplatform (CMArg@Lip), which encapsulates hydrophilic Arg within a ROS‐responsive liposome hollow through hydrophobic interactions. Hydrophobic Ce6 and ML385 are encapsulated within the lipid bilayer of the nanoplatform. As shown in **Scheme** **1**, these nanoplatforms possess a size of ≈100 nm, enabling enhanced permeability and retention‐mediated passive tumor targeting for increased accumulation in tumors. Under light stimulation, Ce6 generates ROS and induces liposome depolymerization, leading to a controlled release of the encapsulated drugs. In the TME, Arg is oxidized by H_2_O_2_ to produce NO. NO exhibits both direct cytotoxic effects against cancer cells and further reacts with ROS generated by Ce6 under light exposure to form the highly lethal ONOO^•^. Simultaneously, ML385 counteracts the intrinsic scavenging capability of cells toward ROS and reactive nitrogen species (RNS). Furthermore, CMArg@Lip exerts immunomodulatory effects within the immunosuppressed TME through three main mechanisms: 1) induction of ferroptosis via CMArg@Lip leads to enhanced ICD; 2) ML385 effectively mitigates the restriction of immune checkpoint blockade on CTLs, as it prevents PDT‐induced upregulation of PD‐L1; 3) CMArg@Lip activates the stimulator of interferon genes (STING) signaling pathway in MDSCs within the TME, resulting in the loss of CTL inhibition and a shift toward an immunostimulatory phenotype that fosters anti‐tumor immunity.

## 2 Result and Discussion

### 2.1 NRF2 Was Upregulated in CCA and Shaped the Antioxidant Microenvironment

Through analysis of the Cancer Genome Atlas Program (TCGA) data, it has been observed that the expression pattern of NRF2 in tumors is different from that in normal tissues. Notably, this difference is particularly significant in abdominal malignancies, including CCA (Figure S1A, Supporting Information). Consistent with these findings, our own collection of CCA specimens revealed heightened levels of NRF2 expression at both the mRNA and protein levels (Figure S1B,C, Supporting Information). Meanwhile, the CCA dataset subjected to PDT underwent enrichment analysis, revealing a noteworthy enrichment of antioxidant genes in the group demonstrating high expression of NRF2 (Figure S1D, Supporting Information). Furthermore, a detailed examination, as exemplified in Figure S1E (Supporting Information), highlighted a collection of target genes that exhibited amplified transcription levels alongside an overexpression of NRF2. Therefore, it is reasonable to speculate that the upregulation of NRF2 in CCA contributes to the preservation of intracellular homeostasis and protects against the development of oxidative stress in TME. Furthermore, it should be noted that ROS generated by PDT can activate NRF2 through the Keap1 pathway.^[^ ^39^ ^]^ Consequently, the persistently high levels of NRF2 activity present in CCA, reactivated by the increased ROS induced by PDT, have the potential to severely compromise the efficiency of PDT. As a result, the combination of PDT with NRF2 inhibitors could prove to be a promising avenue for achieving an optimal synergistic therapeutic effect in the treatment of CCA.

### 2.2 Synthesis and Characterization of CMArg@Lip

**Figure 1:**
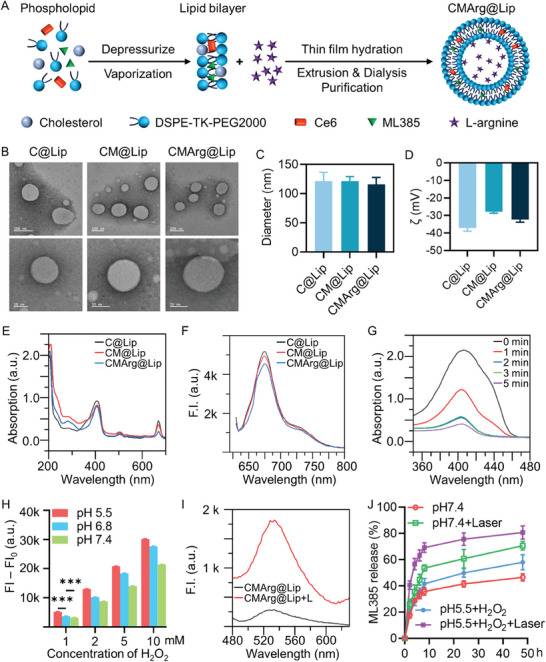
Synthesis and Characterization of CMArg@Lip. A) Schematic synthesis route of CMArg@Lip. B) TEM images of C@Lip, CM@Lip, and CMArg@Lip. Bar: top 100 nm, bottom 50 nm. C,D) C@Lip, CM@Lip, and CMArg@Lip particle sizes and zeta potentials measured by DLS. E) UV–vis absorbance spectra of C@Lip, CM@Lip, and CMArg@Lip. F) Fluorescence spectra of C@Lip, CM@Lip, and CMArg@Lip. G) Detection of ROS generation at different times using DPBF after laser irradiation of CMArg@Lip. H) NO generation of Arg@Lip in an environment with different concentrations of H_2_O_2_ and different pH, detected using DAF‐FM DA. I) Detection of ONOO^−^ using DHR after laser irradiation of CMArg@Lip in an H_2_O_2_ environment. J) Release rate of ML385 from CMArg@Lip in different environments. ^***^, *p* < 0.001.

The CMArg@Lip nanoparticles were prepared using the hydration film method, as illustrated in **Figure** **1**A. The hydrophobic agents, Ce6 and ML385, were loaded into the lipid bilayer through hydrophobic interactions, while the hydrophilic molecule, Arg, was encapsulated within the aqueous cavity of the liposome. The liposomes were characterized by transmission electron microscopy (TEM) and dynamic light scattering (DLS), revealing a distinct spherical structure (Figure 1B). The particle size and ζ potential of all three liposome types were similar, with the CMArg@Lip nanoparticles measuring ≈115.71 ± 11.97 nm in diameter (Figure 1C; Figure S2, Supporting Information). The stable ζ potential values indicated good stability of the liposomes (Figure 1D). The UV–vis absorption spectrum (Figure 1E) demonstrated characteristic peaks for ML385 at ≈282 nm, and for Ce6 at ≈406 and 669 nm, confirming the successful encapsulation of both agents. The drug loading capacity of Arg within CMArg@Lip was determined to be 6.1% through high‐performance liquid chromatography (the drug loading capacity for ML385 and Ce6 were 3.3% and 2.3%, respectively). Fluorescence emission measurements (Figure 1F) further confirmed the successful incorporation of Ce6 into the liposomes, as evidenced by significant fluorescence emitted near 674 nm. Additionally, the liposomes exhibited excellent dispersibility in aqueous solutions.

The production of singlet oxygen (^1^O_2_) by C@Lip, CM@Lip, and CMArg@Lip following irradiation was analyzed through the detection of absorbance using 1,3‐diphenylisobenzofuran (DPBF) (Figure 1G; Figure S3, Supporting Information). A significant reduction in absorbance was observed for C@Lip, CM@Lip, and CMArg@Lip, particularly within the first minute, as compared to H_2_O, indicating rapid ^1^O_2_ generation. Additionally, the production of NO from Arg@Lip under different pH conditions was measured using the DAF‐FM DA probe. The results demonstrated continuous release of NO from Arg@Lip, which was found to be proportional to the concentration of H_2_O_2_ and have better release performance in acidic solutions (Figure 1H; Figure S4, Supporting Information). Following irradiation, NO reacted with locally confined ROS to generate ONOOˉ, thereby enhancing the effectiveness of PDT. The production of ONOOˉ was monitored using dihydrorhodamine 123 (DHR) as a fluorescent probe. As evidenced by the significant absorption peak at 528 nm in CMArg@Lip upon laser irradiation (Figure 1I), the generation of ONOOˉ was confirmed for CMArg@Lip. Moreover, we investigated the drug release kinetics of ML385 under various conditions (Figure 1J). After photostimulation, a higher extent of ML385 release was observed from CMArg@Lip due to the ROS‐responsive TK bond contained in DSPE‐TK‐PEG2000, which constitutes the phospholipid bilayer of the liposome, indicating the desirable ROS‐responsive properties of CMArg@Lip. These findings demonstrate the successful synthesis of CMArg@Lip with ROS‐responsive characteristics, highlighting its potential for efficient ROS and RNS modulation.

### 2.3 CMArg@Lip Were Well Uptake and Effectively Overcome Oxidative Resistance In Vitro

**Figure 2:**
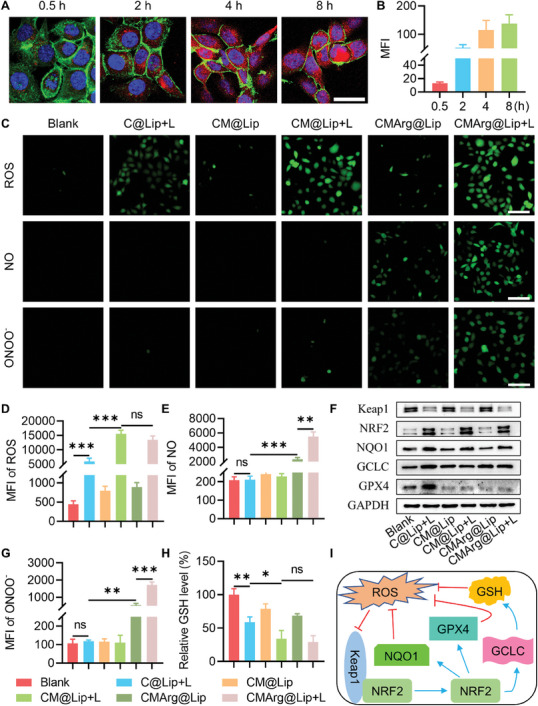
Detection of cellular uptake of CMArg@Lip and its ability to overcome oxidative resistance. A,B) CLSM imaging and mean fluorescence intensity of QBC939 at different times after incubated by CMArg@Lip with 20 µg mL^−1^ (Red: Ce6, Green: cytoskeleton, Blue: nucleus, bar: 20 µm). C,D) Intracellular ROS, NO, and ONOO^−^ production of QBC‐939 were incubated by DCFH‐DA, DAF‐FM DA, or DAX‐J2 PON Green, then observed by fluorescence microscope and detected by flow cytometry after 15 min of different treatments. Bar: 100 µm. E) WB analysis of Keap1, NRF2, NQO1, GCLC, and GPX4 after different treatments. F) The relative level of intracellular GSH was detected after 12 h of different treatments. G) Schematic diagram of NRF2 antioxidant system against ROS. ns, no statistical difference; ^*^, *p* < 0.05; ^**^, *p* < 0.01; ^***^, *p* < 0.001.

Efficient uptake of tumor cells is crucial for the therapeutic effect of nanodrug delivery systems. Liposomes, which have been extensively used in clinical applications for over 50 years, have shown superior efficacy.^[^ ^40^, ^41^, ^42^ ^]^ Through confocal laser scanning microscopy (CLSM), we observed the ingestion of CMArg@Lip by QBC‐939 cells, with a significant increase in uptake observed within 4–8 h (**Figure** **2**A,B). Once inside the tumor cells, the drugs encapsulated in the liposomes began to exert their respective therapeutic actions. The generated ROS and RNS have to do with cellular toxicity. And ML385 interferes with the NRF2 antioxidant system to prevent failure due to the elimination of ROS and RNS. Subsequently, the DCFH‐DA probe was loaded to detect ROS after QBC‐939 cells incubated with different liposomes for 8 h after being treated with or without light (650 nm, 100 mW cm^−2^) for 15 min. Fluorescence images demonstrated that the liposomes added with ML385 produced more ROS in the cells after illumination (Figure 2C; Figure S5, Supporting Information). We used a similar approach to detect the generation of intracellular RNS using DAF‐FM DA and DAX‐J2 PON Green probes. As would be expected, it was found that after adding Arg, the light treatment resulted in the cells producing a large amount of NO and ONOO^•^(Figure 2C; Figure S5, Supporting Information). Theoretically, the addition of Arg may consume ROS produced by Ce6 to a certain extent, but the fluorescence intensity of DCFH‐DA in our detection showed no significant difference between the two groups (CM@Lip + L vs CMArg@Lip + L). This may be due to the fact that the DCFH‐DA is also activated by RNS. The flow cytometry results also showed that CMArg@Lip had the superior ability to produce ROS and RNS in cells relative to other liposomes (Figure 2D; Figure S6, Supporting Information).

We conducted further analysis to investigate the impact of ML385 on the expected outcomes. The activation of NRF2, a transcription factor involved in the cellular response to oxidative stress, is inhibited by Keap1. However, oxidative damage disrupts this inhibition and promotes NRF2 activation, leading to the upregulation of various antioxidant molecules that protect cells from severe damage. Upon exposure to different liposomes, we observed that light treatment degraded Keap1, thereby facilitating NRF2 activation. Notably, only the PDT group (C@Lip+L) demonstrated enhanced NRF2 transcription factor activity, resulting in increased expression of NQO1, GCLC, and GPX4 at both protein and mRNA levels (Figure 2E; Figure S7A,B, Supporting Information). NQO1, regulated by NRF2, plays a crucial role in safeguarding cellular DNA against oxidative damage.^[^ ^43^ ^]^ GPX4, on the other hand, is responsible for neutralizing harmful peroxides,^[^ ^44^ ^]^ while GCLC serves as the rate‐limiting enzyme in GSH synthesis.^[^ ^45^ ^]^ The addition of ML385 significantly suppressed the expression of these genes (Figure 2E; Figure S7A,B, Supporting Information). GSH, a vital non‐enzymatic antioxidant molecule, was found to be consumed in the early stages after light treatment with C@Lip, CM@Lip, and CMArg@Lip. However, over time, GSH levels gradually recovered in the PDT group (C@Lip) and even surpassed the baseline level at 24 h, remaining consistently elevated. Conversely, in the presence of ML385, GSH levels remained consistently low (Figure 2F; Figure S7C, Supporting Information). These findings suggest that intracellular GSH is depleted initially in response to oxidative stress, but its synthesis via GCLC leads to its replenishment and subsequently induces cellular tolerance against oxidative damage. These intricate antioxidant systems within cells serve as critical defense mechanisms against oxidative injury (Figure 2G), and the efficacy of PDT alone is significantly compromised. The addition of ML385‐loaded liposomes, however, overcomes oxidative resistance and maximizes oxidative damage.

### 2.4 CMArg@Lip Promoted Lipid Peroxidation and Doomed Cells Through Ferroptosis

**Figure 3:**
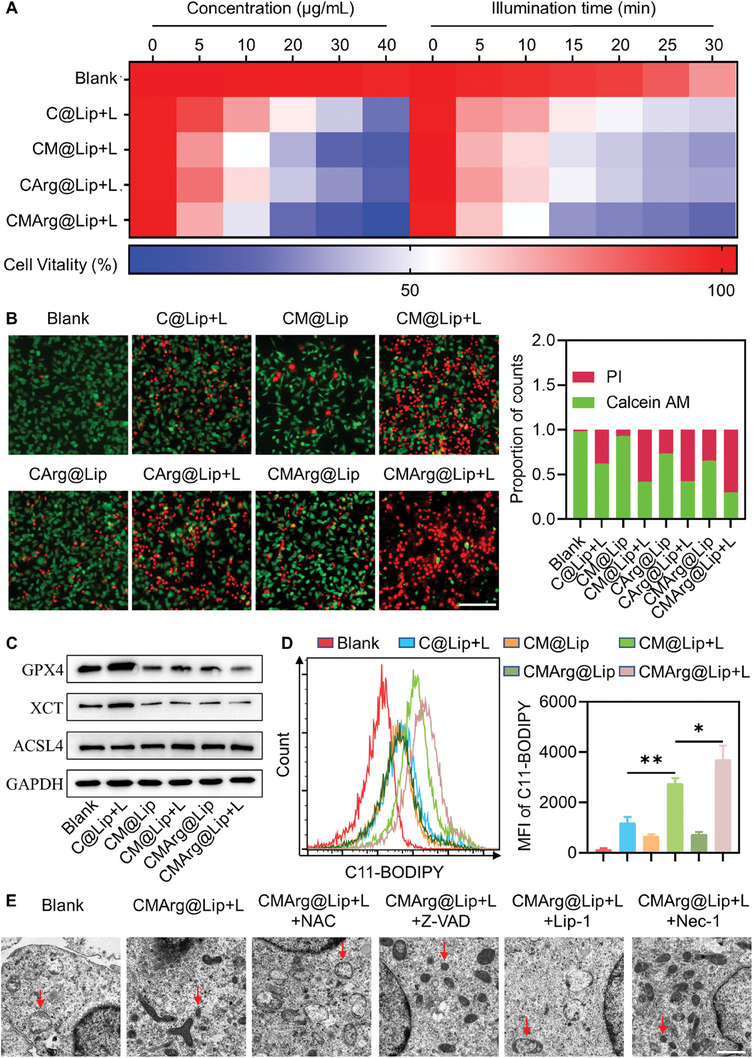
CMArg@Lip promoted lipid peroxidation and doomed cancer cells through ferroptosis. A) The cell vitality of QBC‐939 after different liposomes with light under gradient concentration (fixed light intensity: 650 nm, 100 mW cm^−2^, 15 min) and gradient illumination time (dose: 20 µg mL^−1^ Ce6). B) Fluorescence images of QBC‐939 after different treatments by Calcein‐AM/PI (Green: Calcein‐AM, Red: PI, Bar: 100 µm). The corresponding statistical results are shown on the right. C) WB analysis of ferroptosis core protein GPX4, XCT, ACSL4 after different treatments. D) Flow cytometry analysis of lipid peroxidation in QBC‐939 cells after different treatments by C11‐BODIPY. E) TEM images of QBC‐939 after CMArg@Lip co‐treatment with different programmed cell death inhibitors under illumination (red arrows point to typical mitochondria under different conditions). Bar: 1 µm. ^*^, *p* < 0.05; ^**^, *p* < 0.01.

After confirming that CMArg@Lip with light could produce more ROS and RNS in CC cells, we then verified if its cytotoxicity was significantly improved. Subsequently, the cytotoxicity of CMArg@Lip under light exposure was evaluated and compared to other liposomes carrying the same dose of photosensitizer. Results showed that CMArg@Lip exhibited significantly higher phototoxicity than other liposomes when subjected to the same light conditions (**Figure** **3**A; Figure S8A, Supporting Information). Additionally, Calcein‐AM/PI double staining further demonstrated that the majority of CCA cells pre‐incubated with CMArg@Lip were killed by light exposure (Figure 3B). These findings were also supported by 7‐AAD staining, which indicated cell death (Figure S8B,C, Supporting Information). Overall, the addition of ML385 and arginine reduced the reliance on Ce6 while significantly enhancing the synergistic effect.

Programmed cell death (PCD) is a genetically regulated process characterized by active cell death, which can occur through various pathways such as apoptosis, ferroptosis, and necroptosis.^[^ ^46^, ^47^, ^48^ ^]^ Classical apoptosis initiated by PDT involves the activation of caspase family proteases accompanied by an imbalance in anti‐apoptotic and pro‐apoptotic protein expression.^[^ ^49^ ^]^ This apoptotic pathway can be inhibited by Z‐VAD‐FMK (Z‐VAD). Ferroptosis, another form of PCD, relies on lipid peroxidation and is dynamically regulated by iron, lipoxygenases, and antioxidant systems (particularly GPX4 and GSH).^[^ ^50^, ^51^ ^]^ Ferroptosis can be inhibited by liproxstatin‐1 (Lip‐1). Necroptosis, a regulated form of necrosis, is controlled by phosphorylation of RIPK1, RIPK3, and MLKL, and can be blocked by necrostatin‐1 (Nec‐1).^[^ ^52^ ^]^ The effectiveness of different PCD pathways varies depending on the approach used. Interestingly, gene set enrichment analysis revealed that apoptosis‐related genes were enriched in normal tissues, whereas ferroptosis‐related genes were more abundant in CCA tissues (Figure S9, Supporting Information). This suggests that CCA is resistant to apoptosis but susceptible to ferroptosis, implying that ferroptosis induction may be a more effective strategy for treating cholangiocarcinoma compared to apoptosis inducers.

To further investigate the mechanism by which CMArg@Lip induces PCD after light treatment, QBC‐939 cells were pretreated with various cell death inhibitors and treated with different liposomes. N‐Acetyl‐L‐cysteine (NAC), a potent radical scavenger, served as a positive control. Cell viability and key regulatory proteins associated with different PCD pathways were assessed. After C@Lip and CArg@Lip treatment with light, cell viability was significantly rescued by Z‐VAD (Figure S10A,C, Supporting Information), indicating the involvement of the classical apoptotic pathway. Increased expression of cleaved caspase‐3 and caspase‐9, as well as an elevated ratio of pro‐apoptotic protein Bax to anti‐apoptotic protein Bcl2 (Figure S11A,B,D, Supporting Information), supported the activation of apoptosis in QBC‐939 cells following C@Lip and CArg@Lip treatment with light. However, these findings were not observed in CM@Lip and CMArg@Lip treatments, suggesting that these two treatments primarily induced cell death through mechanisms other than apoptosis. The viability of cells treated with all three liposomes was not restored by Nec‐1, and there were no changes in the levels of phosphorylated RIPK1, RIPK3, and MLKL (Figure S11C,E, Supporting Information), indicating that necrosis was not the main PCD pathway induced by the treatments. Previous studies demonstrated that the addition of ML385 downregulated GPX4 expression, a core regulator of ferroptosis. Based on this observation, it was hypothesized that CM@Lip and CMArg@Lip treatments primarily induced cell death through ferroptosis.

We conducted additional investigations on indicators associated with ferroptosis. Consistent with our suspicions, the ferroptosis inhibitor Lip‐1 effectively restored cell viability after treating cells with CM@Lip and CMArg@Lip followed by light exposure (Figure S10B,D, Supporting Information). Furthermore, Calcein‐AM/PI staining showed a diminished response of QBC‐939 cells preincubated with Lip‐1 to CMArg@Lip upon light exposure (Figure S12A,B, Supporting Information). In ferroptosis, GPX4 and XCT play significant roles in the antioxidant system, while ACSL4 serves as a lipoxygenase that catalyzes lipid peroxidation. We observed a noticeable decrease in the expression of both GPX4 and XCT in QBC‐939 cells treated with ML385‐spiked liposomes, while ACSL4 expression remained unchanged (Figure 3C; Figure S12C, Supporting Information). These findings suggest that CM@Lip and CMArg@Lip with light treatment may directly induce cell membrane oxidation by promoting ROS generation, irrespective of lipoxygenase catalysis. C11‐BODIPY, a fluorescent probe specific to lipid peroxidation, reacts with oxidized species in the cell membrane, resulting in fluorescence at 510 nm (excitation wavelength: 488 nm). Flow cytometry analysis revealed an increase in the mean fluorescence intensity of C11‐BODIPY, which could be reversed by Lip‐1, indicating the occurrence of ferroptosis (Figure 3D; Figure S12D, Supporting Information). Malondialdehyde (MDA), a product of lipid peroxidation, was found to reach the highest levels following treatment with CMArg@Lip (Figure S12E, Supporting Information). Additionally, TEM observations demonstrated mitochondrial membrane wrinkling and loss of cristae, characteristic of ferroptosis, after CMArg@Lip treatment, which was reversed by Lip‐1 (Figure 3E). These lines of evidence suggest that the introduction of ML385 shifted drug‐loaded liposome‐induced cell death from apoptosis to ferroptosis.

### 2.5 CMArg@Lip Triggered Potent ICD Effects and Promoted DC Cell Maturation In Vitro

Damage‐associated molecular patterns (DAMP), including calreticulin (CRT), high mobility group protein 1 (HMGB1), the heat shock protein (HSP) family, adenosine triphosphate (ATP), and others, are released or displayed when cancer cells are stimulated or destroyed to help immune cells identify and eliminate cancer cells.^[^ ^53^ ^]^ This biological event is known as immunogenic cell death (ICD). Although prior research has shown that PDT is effective in triggering ICD, the degree of ICD produced by various cell death pathways varies. Consistent with previous studies, both CRT expression on the cytomembrane of QBC‐939 cells (Figure S13A,D, Supporting Information) and HSP70 expression (Figure S13C,F, Supporting Information) were considerably enhanced after treatment with drug‐loaded liposomes, whereas HMGB1 levels in the nucleus were decreased (Figure S13B,E, Supporting Information), particularly in the CMArg@Lip with light treatment. Despite this, total intracellular CRT was essentially unchanged, although HSP70 expression was increased (Figure S14A,B,D, Supporting Information), which was accompanied by a decrease in intracellular HMGB1 (Figure S14A,C, Supporting Information) and an increase in extracellular HMGB1 (Figure S14E, Supporting Information) and ATP (Figure S14F, Supporting Information), confirmed the activation of ICD with potential anti‐tumor immunity. To further confirm this, we repeated the treatment of QBC‐939 and co‐cultured it proportionally with immature DC cells added to the original medium (Figure S15A, Supporting Information). And found that the proportion of mature DC cells was significantly higher in the treatment group containing ML385 than in the PDT alone group and that the effect caused by CMArg@Lip with light treatment was slightly better than in the CM@Lip with light treatment group (Figure S15B, Supporting Information). These suggest that treatment containing ML385 induced a shift from apoptosis to ferroptosis in QBC‐939, enhancing the ICD effect which was further amplified by increased oxidative stress with the addition of arginine.

### 2.6 CMArg@Lip Relieved PDT‐Induced Immune Resistance

PDT has been shown to increase the expression of PD‐L1 in previous studies.^[^ ^19^, ^54^, ^55^ ^]^ One well‐known pathway responsible for this increase is the hypoxia‐inducible factor 1α (HIF1α) pathway, which is activated as PDT consumes oxygen and creates a tumor‐hypoxic microenvironment. HIF1α directly upregulates PD‐L1 at the transcriptional level. However, our curiosity was piqued when we observed a significant increase in PD‐L1 even after improving hypoxia (Figure S16A,B, Supporting Information). Another transcription factor, NRF2, accumulates after oxidative stress caused by PDT (Figure S16A,B, Supporting Information). Recent studies have suggested that NRF2 acts as a transcription factor for PD‐L1.^[^ ^27^ ^]^ Thus, we aimed to investigate the potential regulatory pathway of NRF2/PD‐L1 after PDT in CCA. As expected, we detected an elevation of NRF2 after PDT, showing dose dependence. Additionally, PD‐L1 exhibited consistent variation and could be reversed by ML385, a NRF2 inhibitor (Figure S16C–F, Supporting Information). Similarly, bardoxolone, a NRF2 activator, had a PD‐L1‐inducing effect similar to PDT. Knockdown of NRF2 also effectively inhibited PDT‐induced PD‐L1 expression (Figure S16G,H, Supporting Information). However, the knockdown of NRF2 alone showed minimal impact on PD‐L1 expression in QBC939 cells. Furthermore, analysis of TCGA‐CHOL and GSE132305 datasets revealed a low correlation coefficient between NRF2 and PD‐L1 (Figure S17A,B, Supporting Information). Nevertheless, NRF2 was strongly positively correlated with PD‐L1 expression in another dataset, GSE84756, which considered PDT stimulation (Figure S17C, Supporting Information). Based on these findings, it is inferred that in CCA, the activation of the NRF2/PD‐L1 pathway depends on the stimulation from PDT. In other words, the innate high NRF2 levels activated by PDT further enhance immunosuppression, thus hindering anti‐tumor immunotherapy. This is where ML385 demonstrates its diverse functions, mitigating these issues by blocking the transcriptional activity of NRF2.

**Figure 4:**
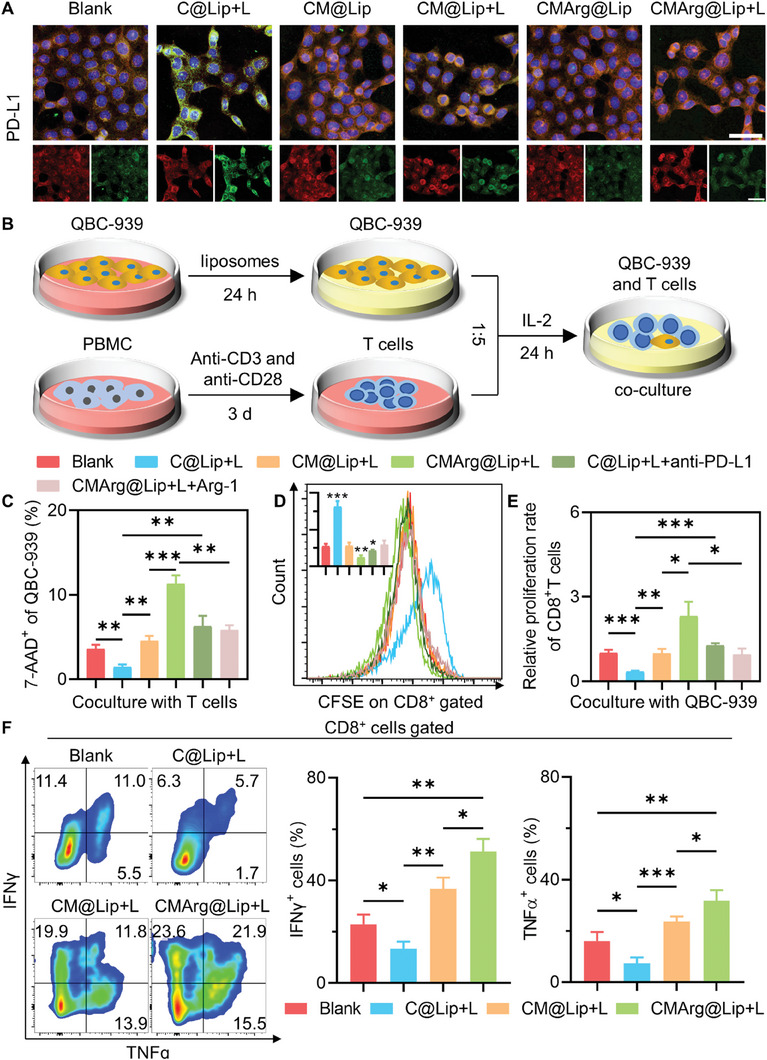
CMArg@Lip relieved PD‐L1 inhibition of T cell proliferation and function. A) Immunofluorescence images of QBC‐939 with different treatments after staining of PD‐L1 on the surface (Green: PD‐L1, Red: cytomembrane, Blue: nucleus, Bar: 50 µm). B) Schematic representation of induced T cells co‐cultured with treated QBC‐939. C) Cell dead detection of treated QBC‐939 by 7‐AAD staining after co‐culture with induced T cells. D,E) CFSE pre‐labeled induced T cells were co‐incubated with treated QBC‐939 for 24 h before flow cytometry to detect changes in CFSE fluorescence intensity (the top left corner showed the mean fluorescence intensity of CFSE) and calculate the relative proliferation rate of CD8^+^ T cells. F) Flow cytometry detection of the ratio of IFNγ^+^ and TNFα^+^ cells in CD8^+^ cells after 24 h co‐incubation of induced T cells with treated QBC‐939. ^*^, *p* < 0.05; ^**^, *p* < 0.01; ^***^, *p* < 0.001.

Therefore, the subsequent step involved verification of whether the inclusion of ML385 in the therapy could effectively attenuate immune checkpoint activation induced by PDT. Upon subjecting QBC‐939 to various liposomes, it was observed that PDT treatment alone led to a marked escalation in both total PD‐L1 expression (Figure S18A, Supporting Information) and membrane‐bound PD‐L1 (**Figure** **4**A; Figure S18B,C, Supporting Information). However, this effect was successfully reversed when combined with ML385, while the addition of arginine did not exert any influence on the outcomes. Immune checkpoints play a crucial role in modulating the antitumor activity of CTLs, which primarily comprise CD8^+^ T cells. In order to evaluate the efficacy of ML385 in suppressing immunological checkpoints activated by PDT, QBC‐939 cells treated with various liposomes were co‐incubated with stimulated T cells pre‐labeled with CFSE, a living cell fluorescent dye whose intensity diminishes as cells undergo proliferation (Figure 4B). When compared to PDT pretreatment alone, CM@Lip + L and CMArg@Lip + L induced more severe damage to QBC‐939 (Figure 4C; Figure S18D, Supporting Information), which could be attributed to enhanced proliferation (Figure 4D,E) and activation (Figure 4F) of CD8^+^ T cells. In other words, the inclusion of ML385 counteracted the detrimental effects of PDT‐induced PD‐L1 activation. Furthermore, the trophic impact of arginine on T cells was underscored by the finding that the CMArg@Lip+L pretreatment group exhibited an augmented CD8^+^ T cell activation effect, which could be inhibited via arginase‐1 (Arg‐1). These findings indicate that ML385 effectively rectified PDT‐induced PD‐L1 overexpression, and that arginine supplementation further enhanced CD8^+^ T cell‐mediated antitumor immunity.

### 2.7 CMArg@Lip Alleviated MDSC‐Induced Intrinsic Immune Resistance

**Figure 5:**
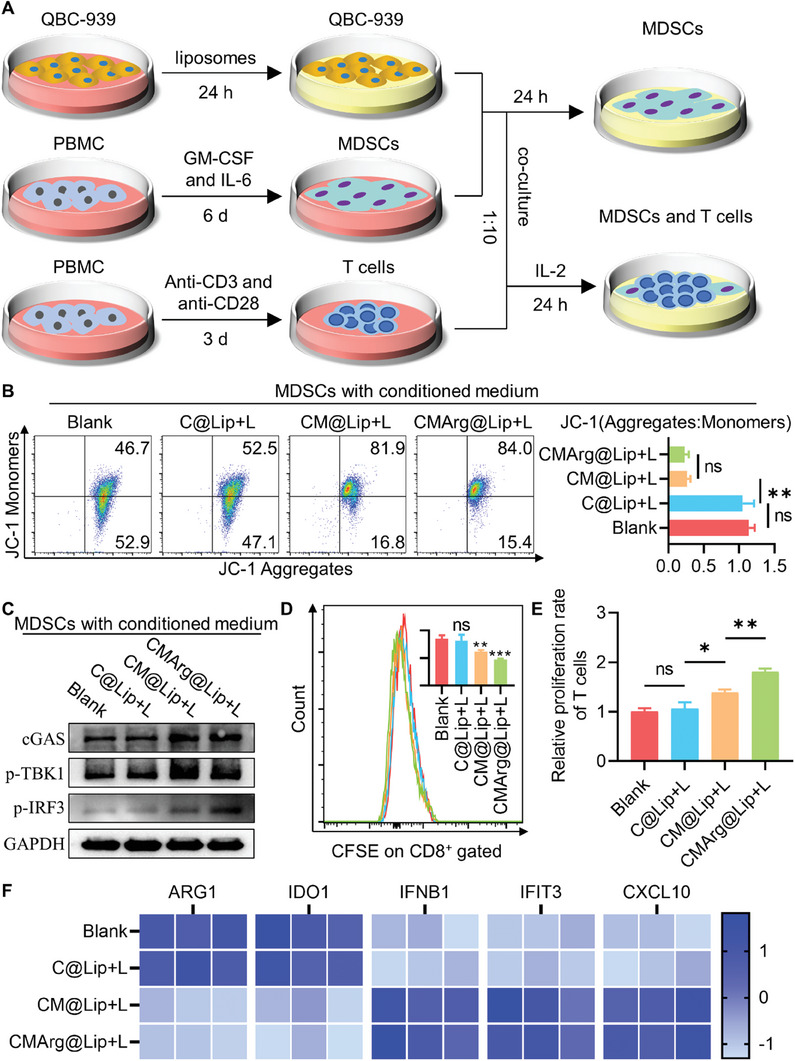
CMArg@Lip activated the Sting pathway in MDSC. A) Diagram of induced MDSCs cultured using conditioned medium which cultured QBC‐939 after receiving treatment and co‐cultured with induced T cells in proportion. B) Flow cytometry detection of JC‐1 staining of induced MDSCs treated with different conditioned media. C) WB analysis of sting pathway‐associated protein cGAS, p‐TBK1, and p‐IRF3 in induced MDSCs cultured with different conditioned media. D,E) CFSE pre‐labeled induced T cells were co‐incubated with MDSCs in conditioned media for 24 h before flow cytometry and calculated the relative proliferation rate of CD8^+^ T cells. F) q‐PCR analysis of changes in genes suppressing (ARG1, IDO1) and promoting (IFNB1, IFIT3, CXCL10) antitumor immune‐related genes in MDSC cultured in conditioned media. ns, no statistical difference; ^*^, *p* < 0.05; ^**^, *p* < 0.01; ^***^, *p* < 0.001.

In fact, there is a significant infiltration of immunosuppressive cells within TME, which hinders the effectiveness of CTLs. One such group of them was known as MDSCs. Previous research has demonstrated that blocking NRF2 signaling in MDSCs can activate the STING signaling pathway, leading to a reversal of their immunosuppressive phenotype.^[^ ^30^ ^]^ Thus, our objective was to investigate whether CMArg@Lip has an impact on the functional phenotype of MDSCs (**Figure** **5**A). We observed an increase in mitochondrial membrane depolarization in MDSCs treated with a conditioned medium containing ML385, as evident from a higher proportion of JC‐1 monomers (Figure 5B). This indicates the release of mitochondrial DNA, resulting in the activation of the STING pathway. Our study also confirmed elevated expression of cGAS, p‐TBK1, and p‐IRF3 in MDSCs treated with conditioned medium containing ML385 (Figure 5C; Figure S19, Supporting Information), which are markers of cGAS‐STING pathway activation.

Activation of the STING pathway led to reduced expression of immunosuppressive genes ARG1 and IDO1 (Figure 5F), along with increased expression of interferon‐related genes IFNB1, IFIT3, and CXCL10 in MDSCs (Figure 5G). These findings suggest a loss of immunosuppressive phenotype in MDSCs and a shift toward promoting anti‐tumor immunity. To confirm this effect, we conducted co‐culture experiments using induced MDSCs and induced T cells (Figure 5A). In the presence of a conditioned medium containing ML385, inhibition of CD8^+^ T cell proliferation by MDSCs was alleviated, and as previously observed, CD8^+^ T cell proliferation became more active in the CMArg@Lip pretreatment system due to the arginine nutrition effect (Figure 5D,E). Collectively, these findings provide evidence that CMArg@Lip can effectively reverse the immunosuppressive activity of MDSCs and further enhance CTL‐mediated anti‐tumor immunity within the TME.

### 2.8 CMArg@Lip Suppressed CCA In Vivo

**Figure 6:**
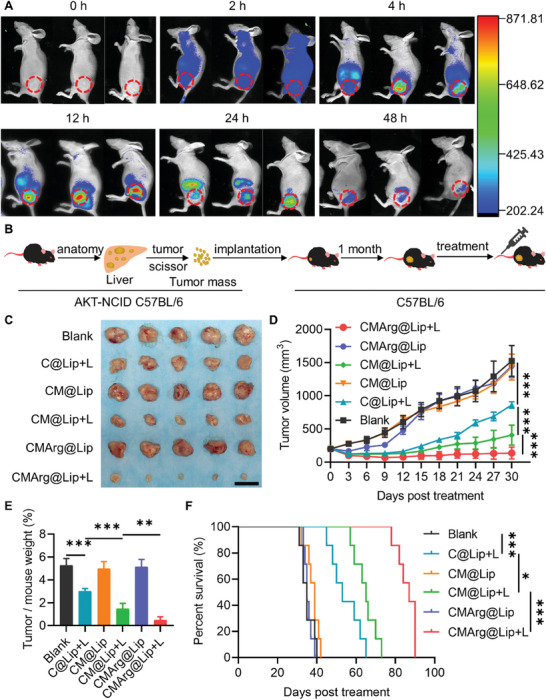
CMArg@Lip accumulated in tumors in vivo and effectively inhibited tumor growth. A) In vivo imaging at different time points after injecting CMArg@Lip into the tail vein of tumor‐bearing Balb/c nude mice (*n* = 3). B) Construction of a tumor‐bearing model of C57BL/6J mouse cholangiocarcinoma. C) Tumors were collected from mice after 30 days of different treatments (Bar: 2 cm, *n* = 5). D) Tumor volume growth in 30 days in mice receiving different treatments (*n* = 5). E) Ratio of tumor weight to mouse weight at 30 days in mice receiving different treatments (*n* = 5). F) Survival curves of tumor‐bearing mice receiving different treatments (*n* = 7). ^*^, *p* < 0.05; ^**^, *p* < 0.01; ^***^, *p* < 0.001.

Subsequently, drug‐loaded liposomes were administered in vivo and their therapeutic outcomes were evaluated. Balb/c nude mice bearing QBC‐939 tumors were intravenously injected with CMArg@Lip, which exhibited gradual accumulation at the tumor site and detectability after 48 h (**Figure** **6**A). Isolated organ imaging confirmed that CMArg@Lip predominantly localized within the tumor (Figure S20, Supporting Information), indicating its exceptional tumor accumulation and retention abilities.

Considering the potent immunomodulatory effects of CMArg@Lip and the absence of immunocompetent mouse models for CCA, a homologous transplant CCA model was developed to assess the in vivo therapeutic efficacy of CMArg@Lip. Figure 6B illustrates the construction process of the AKT/Notch internal domain (NCID) CCA model and the homologous transplant tumor model. Successful establishment of the tumor model was confirmed by HE and CK7 staining (a CCA marker) (Figure S21, Supporting Information). Once the transplanted tumors reached a volume of 200 mm^3^, various drug‐loaded liposomes were administered, and the tumor area was subjected to 15‐min irradiation with or without light after 12 h. In all subgroups, CMArg@Lip with light therapy resulted in a significant reduction of tumor growth (Figure 6C–E) and increased survival (Figure 6F) in mice. Moreover, HE and Ki‐67 staining (Figure S22A, Supporting Information) demonstrated a significant suppression of tumor proliferation by CMArg@Lip+L therapy. Of note, the upregulation of the neovascularization marker CD31 (Figure S22B, Supporting Information) in arginine‐containing therapies was intriguing. This advantageous phenomenon in PDT‐based combination therapy was attributed to the production of NO by arginine, which stimulates neovascularization and facilitates oxygen diffusion, ultimately rectifying hypoxia. The use of arginine confirmed the reversal of the hypoxic microenvironment, as evidenced by the reduced fluorescence intensity of HIF‐1α and hypoxyprobe staining (Figure S22C, Supporting Information). ML385 consistently prevented the activation of antioxidants induced by PDT in vivo, as indicated by NRF2 and GPX4 staining (Figure S22D, Supporting Information). These findings demonstrate that CMArg@Lip with light therapy effectively counteracts the PDT‐induced antioxidant and hypoxic conditions within tumors in vivo, thereby maximizing therapeutic efficacy.

### 2.9 CMArg@Lip Reversed the Immunosuppressive TME In Vivo

**Figure 7:**
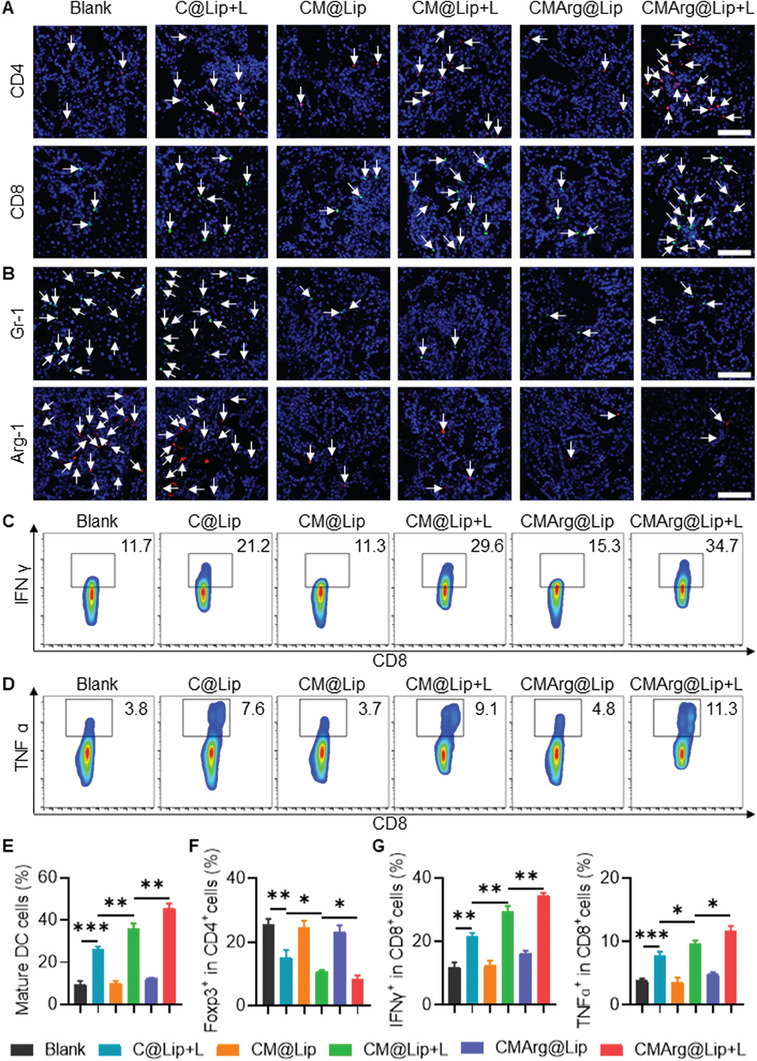
CMArg improved the immunosuppressive microenvironment in vivo and activated anti‐tumor immunity. A) Tumors were collected from tumor‐bearing mice after receiving different treatments for CD4 and CD8 immunofluorescence staining. B) Immunofluorescence staining of MDSCs marker (Gr1 and Arg‐1) in tumor‐bearing mice collected after receiving different treatments. Bar: 100 µm. C,D) Flow cytometry detected functional activation (IFNγ^+^ and TNFɑ^+^) of tumor entry CD8^+^ T cells in tumor‐bearing mice after treatment. E–G) Flow cytometry analysis of tumor‐infiltrating mature DC cells, Treg cells (Foxp3^+^CD4^+^), and IFNγ^+^CD8^+^ or TNFɑ^+^ CD8^+^ cells ratio in tumor‐bearing mice receiving different treatments. ^*^, *p* < 0.05; ^**^, *p* < 0.01; ^***^, *p* < 0.001.

Furthermore, we evaluated the efficacy of drug‐loaded liposomes in reducing immunosuppression and inducing antitumor immunity in vivo. Tumor‐infiltrating dendritic cells (DCs), exhibited increased presence due to the upregulation of CRT^+^ cells (Figures S23A,D and S24, Supporting Information). The in vivo experiment demonstrated that CMArg@Lip with light treatment consistently produced an ICD effect, which aligned with our in vitro findings. This upregulation facilitated enhanced tumor antigen delivery and subsequent recruitment of CTLs. Immunofluorescence images confirmed that CMArg@Lip treatment generated the highest anti‐tumor immunity, evidenced by an increase in the number of CD4^+^ and CD8^+^ cells infiltrating the tumor (**Figure** **7**A; Figure S25A,B, Supporting Information). Moreover, the administration of ML385 effectively prevented PD‐L1 activation induced by PDT in vivo (Figure S23B,E, Supporting Information). The therapy of CMArg@Lip + L successfully regulated the number of Gr1^+^ cells and the expression of the immunosuppressive phenotype marker Arg‐1^+^ on MDSCs within TME, achieving maximal suppression, as represented in Figure 7B and Figure S25C,D (Supporting Information). Foxp3, a marker gene for regulatory T cells (Tregs), which has been implicated in restricting the cytotoxic activity of CTLs against tumors, showed no significant change in infiltration after treatment with drug‐loaded liposomes (Figure S23C,F, Supporting Information). However, the percentage of Tregs within the total CD4^+^ cell population gradually reduced (Figure 7F; Figure S26, Supporting Information), suggesting a potential decrease in Treg function during CMArg@Lip with light treatment. Notably, the increase in IFN^+^ CD8^+^ cells (Figure 7C) and TNF^+^ CD8^+^ cells (Figure 7D,G) within the TME provided direct evidence, supporting the substantial immunotherapeutic efficacy of CMArg@Lip with light treatment under the dual effects of relieving immunosuppression and activating antitumor immune responses.

### 2.10 Biosafety Evaluation of CMArg@Lip

Finally, in order to assess the biosafety of CMArg@Lip, both in vitro and in vivo experiments were conducted. This was necessary despite previous clinical evidence supporting the safety of drug‐laden liposomes. In the in vitro study, graded doses of CMArg@Lip were combined with mouse blood. Remarkably, even at high concentrations, no hemolysis was observed (Figure S27, Supporting Information). Furthermore, during the in vivo study, all groups of mice exhibited steady weight gain throughout the therapy period, indicating minimal systemic toxicity associated with CMArg@Lip treatment (Figure S28, Supporting Information). Histopathological examination of normal tissues stained with HE stain revealed no damage when utilizing twice the dose of drug‐loaded liposomes in mice (Figure S29A, Supporting Information). Moreover, there were no significant alterations in terms of blood cell counts or biochemical indices among the various treatment groups, all of which remained within normal ranges (Figure S29B, Supporting Information). These findings collectively demonstrate the exceptional biosafety profile of CMArg@Lip, both in vitro and in vivo.

## 3 Conclusion

We have successfully developed a nanoplatform that enables the implementation of integrated PDT‐gas therapy and supports anti‐tumor immunomodulation. One of the key mechanisms involved in this process is the inhibition of NRF2. CMArg@Lip exhibited superior performance in inducing oxidative damage, by disrupting the robust antioxidant capacity of cancer cells and promoting the generation of ROS and RNS during treatment. Consequently, ferroptosis was induced in the cancer cells. In terms of immune microenvironment regulation, CMArg@Lip demonstrated significant effects on ICD, leading to enhanced DC maturation and reversal of PDT‐induced immune tolerance. Furthermore, through the regulation of NRF2, MDSC, an immunosuppressive cell present in the CCA microenvironment, was reprogrammed into a phenotype that promotes the anti‐tumor function of CTLs via activation of the STING pathway. In summary, CMArg@Lip effectively overcomes the challenges of tissue hypoxia and antioxidant defenses encountered by PDT, thereby achieving the integration of PDT‐gas immunotherapy.

## 4 Experimental Section

### Materials

Soy Lecithin (SPC), cholesterol, and DSPE‐TK‐PEG2000 were obtained from Xi'an Ruixi Biological Technology Co., Ltd (Xian, China). Chlorin e6 (Ce6) was provided by Solarbio LIFE SCIENCES (Beijing, China). ML385 was brought from MedChemExpress (MCE) (Shanghai, China). L‐Arginine (Arg) was purchased from GL Biochem Ltd (Shanghai, China). All cell fluorescence probes and detection kits were purchased from Beyotime (Shanghai, China). And all biological inhibitors were purchased from MedChemExpress (MCE) (Shanghai, China). Ultrapure mili‐Q water was used in all experiments.

### Synthesis and Characterization of Drug‐Loaded Liposome

Liposomes were prepared by thin‐film hydration method. Briefly, SPC, cholesterol, and DSPE‐TK‐PEG2000 solution (25 mg mL^−1^ in chloroform) were mixed with a mass ratio of 65:23:12. For Ce6, ML385, and L‐Arg loaded liposome (CMArg@Lip), Ce6 and ML385 were dissolved in chloroform and added into lipid solution. The lipid film was obtained by solvent evaporation through N_2_ flow and more than 2 h rotary evaporator. The thin film was hydrated with water containing Arg with the concentration of 2 mg mL^−1^ at 60 °C for 30 min to pre‐dispersion. The suspension was probe sonicated for two 5 min cycles by ultrasonic cell disruptor, CMArg@Lip was obtained. Ce6‐loaded liposome (C@Lip) and Ce6&ML385 loaded liposome (CM@Lip) were obtained through a similar procedure as mentioned above. The liposomes were characterized by transmission electron microscope (TEM, TF20, FEI company, USA), nanoparticle potentiostat (NanoBrook 90plus PALS, Brookhaven, USA), and UV–vis spectrophotometry (TU‐1810, Beijing General Analytical Instrument, China). Fluorescence spectrophotometers of the C@Lip, CM@Lip, and CMArg@Lip were analyzed via a Fluoromax‐4 spectrofluorometer (Horiba Jobin Yvon Inc.) under 600 nm excitation. The encapsulation efficiency (EE) and the drug loading capacity (DL) of ML385 were calculated by high‐performance liquid chromatography (HPLC, Agilent Technologies Inc., 1100S, USA) with a UV–vis detector at a wavelength of 282 nm. The EE and DL of Ce6 and Arg were analyzed by UV–vis spectrophotometer or HPLC, as follows: C@Lip (Ce6, EE: 93%, DL: 4.65%), CM@Lip (Ce6, EE: 94%, DL: 2.4%; ML385, EE: 93%, DL: 3.4%), and CMArg@Lip (Ce6, EE: 92%, DL: 2.3%; ML385, EE: 94%, DL: 3.3%; L‐Arg, EE: 76%, DL: 6.1%).

### Detection of Singlet Oxygen Generation

The singlet oxygen was evaluated by detecting the declined absorption of 1,3‐diphenylisobenzofuran (DPBF) at 416 nm after 655 nm irradiation for a period of time. Various liposomes were dispersed in H_2_O, and 10 µg mL^−1^ DPBF/DMSO solution was added into H_2_O, C@Lip, CM@Lip, and CMA@Lip containing Ce6 with the concentration of 5 µg mL^−1^. Subsequently, the solution above was exposed under 655 nm laser irradiation (0.2 W, 5 min), and then was analyzed by the UV–vis spectrophotometer.

### Measurement of NO Generation by DAF‐FM DA Probe

The production of NO was detected using a sensitive fluorescent probe DAF‐FM DA. Typically, 200 µL of Arg@Lip with a concentration of 400 µg mL^−1^ in PBS (pH 5.5, 6.8, and 7.4) and various H_2_O_2_ (0, 1, 2, 5, and 10 mm) were added, respectively. Then fluorescence intensity was detected after adding DAF‐FM DA.

### The Test of ONOO ¯

In the acidic environment, ONOO ¯ was generated in the present NO and ROS. To evaluate the production of ONOO ¯, dihydrorhodamine123 (DHR) was employed. The suspensions containing 0.1 mm DHR, 10 mm H_2_O_2_, and CMArg@Lip in 0.6 mL PBS were irradiated for 5 min with a 655 nm laser at a power of 0.2 W. Then, the suspensions were detected by UV–vis spectroscopy.

### The Drug Release Behavior Study

To study the ML385 release, a solution of CMArg@Lip was dialyzed against PBS with different pH values (5.5 and 7.4) containing 0.5% between 80 at 37 °C with a shake hand in a tube. At certain porins (2, 4, 6, 8, 24, 48 h), the solution outside the dialysis tube was collected and the equal fresh corresponding PBS was added. The amounts of ML385 were measured by HPLC, the cumulative release rates were further calculated.

### Cell Lines and Animals

The CCA cell line (QBC‐939 cells) was purchased from Shanghai Cell Bank of Chinese Academy of Sciences (Shanghai, China), and cultured in RPMI‐1640 medium (Gibco) containing 10% FBS (Hycezbio, Wuhan, China) and 1% Penicillin‐Streptomycin at 37 °C in 5% CO_2_. And 6‐week‐old female Balb/c nude mice and C57BL/6J mice were purchased from the Vital River Laboratory Animal Technology Co. (Beijing, China) and the Beijing HFK Bioscience Co. (Beijing, China) were neutrally housed at Tongji Medical College Animal Experimentation, and the experiments were conducted according to the guidelines established by the Ethics Committee of Tongji Medical College, HUST, Wuhan, China ([2020] IACUC nunber:2910).

### Data Mining of TCGA and GEO Databases

CCA mRNA‐seq data TCGA‐CHOL dataset downloaded from The Cancer Genome Atlas Program (https://cancergenome.nih.gov/), GSE132305 and GSE84756 datasets downloaded from Gene Expression Omnibus (https://www.ncbi.nlm.nih.gov/geo/). NRF2 mRNA expression differences and survival analysis were performed using GEPIA (http://gepia.cancer‐pku.cn). And enrichment analysis of ferroptosis and apoptosis gene sets using GSEA.

### Cell Uptake Test

The exponential growth of QBC‐939 cells was grown overnight in 12‐well plates with pre‐placed coverslips. And then the culture medium containing 20 µg mL^−1^ CMArg@Lip was changed to continue the culture. The medium was removed and washed with PBS at 0.5, 2, 4, and 8 h, respectively. Next, the cells were fixed with 4% paraformaldehyde for 20 min and the cell membrane was disrupted with 0.1% Triton X‐100 in PBS for 5 min. Subsequently stained with FITC‐Phalloidin for 20 min and Hoechst for 4 min. PBS was used to wash three times before each new reagent was changed. Finally, a laser confocal microscope (Nikon, Japan) was used to observe and obtain images.

### Intracellular ROS and RNS Detection

After attachment of QBC‐939 cells inoculated in six‐well plates, the medium containing different drug‐loaded liposomes (20 µg mL^−1^) was switched to incubate for 6 h. The DCFH‐DA, DAF‐FM DA, or DAX‐J2 PON Green (Beyotime, Shanghai, China) fluorescent probes were then loaded according to the instructions. After incubation in the dark or 650 nm light for 15 min, cells were observed using a fluorescence microscope or collected for flow cytometric detection.

### Intracellular GSH Detection

Intracellular GSH content was determined using the kit according to the manufacturer's instructions. Attached cells in six‐well plates were incubated with different drug‐loaded liposomes for 6 h followed by light (650 nm) for 15 min and incubated using dark as the control. The treated cells were collected, the supernatant was removed by centrifugation, and the protein removal reagent S solution was added and fully vortexed. Cells were rapidly frozen and thawed twice using liquid nitrogen and a 37 °C water bath and then left to rest for 5 min. After centrifuging and collecting, the supernatant was mixed with a working solution to detect A412 by Microplate Reader.

### Cell Viability Assay

Cell viability of QBC‐939 after different treatments was detected by CCK8 assay. After culturing 10 000 cells overnight in 96‐well plates, the medium containing different drug‐loaded liposomes was replaced with light treatment (650 nm, 100 mW cm^−2^, 15 min) after 6 h, and the culture was continued for 24 h. Subsequently, the original medium was removed and the medium containing CCK8 was added and incubated for 1 h, and A450 was detected by Microplate Reader.

### Western Blot

The western blot was performed according to a standard procedure. Protein samples were extracted using a mixture of RIPA and protease inhibitors, followed immediately by normalization of sample protein concentrations using the BCA. After SDS‐PAGE, the protein is transferred to the NC membrane. The non‐specific binding site was then blocked for 1 h to allow for primary antibody incubation overnight at 4 °C. The next day, after incubation with the secondary antibody, the protein was visible by the ECL luminescent substrate. The following antibodies were used: anti‐Keap1 (Proteintech, 10503‐2‐AP), anti‐NRF2 (Proteintech, 16396‐1‐AP), anti‐NQO1 (Bioswamp, PAB43946), anti‐GCLC (Bioswamp, PAB30447), anti‐GPX4 (Proteintech, 67763‐1‐Ig), anti‐XCT (Abmart, T57046), anti‐ACSL4 (Proteintech, 66617‐1‐Ig), anti‐cGAS (Bioswamp, PAB31938), anti‐pTBK1 (Bioswamp, PAB43634‐P), anti‐pIRF3 (Bioswamp, PAB43644‐P), anti‐caspase9 (Proteintech, 10380‐1‐AP), anti‐Bax (Proteintech, 60267‐1‐Ig), anti‐Bcl2 (Affinity, BF9103), anti‐RIPK1 (ABclonal, A19580), anti‐pRIPK1 (ABclonal, AP1115), anti‐RIPK3 (ABclonal, A5431), anti‐pRIPK3 (ABclonal, AP1260), anti‐MLKL (Biodragon, BD‐PT2788), anti‐pMLKL (Abcam, 187 091), anti‐CRT (Proteintech, 27298‐1‐AP), anti‐HMGB1 (Proteintech, 1029‐1‐AP), anti‐HSP70 (Proteintech, 66183‐1‐Ig), anti‐HIF1ɑ (Proteintech, 66703‐1‐Ig), anti‐PD‐L1 (Bioswamp, PAB45988), anti‐GAPDH (Proteintech, PAB35820), anti‐Vinculin (Proteintech, 66305‐1‐Ig).

### Quantitative Real‐Time PCR

The primers used in qPCR were designed as follows: NFE2L2 forward 5′‐AGTCCAGAAGCCAAACTGACAGAAG −3′, reverse 5′‐GGAGAGGATGCTGCTGAAGGAATC‐3′; NQO1 forward 5′‐CCACCTCCTGAGTTCAAGCGATTC‐3′, reverse 5′‐GAGTTCAAGACCAGCCTGACCAAC‐3′; GPX4 forward 5′‐ATGGTTAACCTGGACAAGTACC‐3′, reverse 5′‐GACGAGCTGAGTGTAGTTTACT‐3′; GCLC forward 5′‐TGTCCGAGTTCAATACAGTTGA‐3′, reverse 5′‐ACAGCCTAATCTGGGAAATGAA‐3′; CD274 forward 5′‐GCTGCACTAATTGTCTATTGGG‐3′, reverse 5′‐ CACAGTAATTCGCTTGTAGTCG‐3′; ARG1 forward 5′‐GGACCTGCCCTTTGCTGACATC‐3′, reverse 5′‐TCTTCTTGACTTCTGCCACCTTGC‐3′; NOS2 forward 5′‐ CAGGGTGGAAGCGGTAACAAAGG‐3′, reverse 5′‐CCTGCTTGGTGGCGAAGATGAG‐3′; IDO1 forward 5′‐GCCCTTCAAGTGTTTCACCAAATCC‐3′, reverse 5′‐ GGGTTGCCTTTCCAGCCAGAC‐3′; IFNB1 forward 5′‐ GAAGGAGGACGCCGCATTGAC‐3′, reverse 5′‐ACAATAGTCTCATTCCAGCCAGTGC‐3′; IFIT3 forward 5′‐TACGCCTGGGTCTACTATCACTTGG‐3′, reverse 5′‐CACTTCAGTTGTGTCCACCCTTCC‐3′; CXCL10 forward 5′‐ATTCCTGCAAGCCAATTTTGTCCAC‐3′, reverse 5′‐TGATGGCCTTCGATTCTGGATTCAG‐3′.

### Calcein‐AM/PI Staining

Calcein‐AM/PI staining was used to differentiate between QBC‐939 cells that survived or died after receiving different treatments. After the QBC‐939 cells in six‐well plates received different treatments, the medium was removed, washed gently with PBS, and treated with Calcein‐AM and PI fluorescent dye in the dark for 30 min. Subsequently, images were observed and acquired using a fluorescent microscope, and the ratio of live to dead cells was counted.

### 7‐AAD Staining

7‐AAD fluorescent probe was used to detect the death of QBC‐939 cells after receiving different treatments. After treatment, the cells floating in the culture medium need to be taken seriously and collected together. Cell death was detected using flow cytometry after 10 min incubation with the 7‐AAD probe.

### C11‐BODIPY Staining

The C11‐BODIPY probe was used to detect the extent of lipid peroxidation in QBC‐939 cell membranes after treatment. After being collected in six‐well plates, the treated QBC‐939 cells were incubated for 30 min by loading the C11‐BODIPY probe. The flow cytometry was used to detect the fluorescence intensity of C11‐BODIPY (510 nm).

### Immunofluorescence Staining

Immunofluorescence staining was used to detect changes in the distribution and content of proteins within cells. QBC‐939 cells were grown in 12‐well plates with pre‐placed coverslips overnight for different treatments. The cells were fixed with 4% paraformaldehyde for 20 min and the cell membrane was disrupted with 0.1% Triton X‐100 in PBS for 5 min (detection of proteins on cell membranes without Triton X‐100). Afterward, primary antibodies were added and incubated overnight at 4 °C. And all coverslips were treated with goat serum for 1 h prior to incubation with antibodies to eliminate non‐specific binding. The next day, after removing the primary antibody, a secondary antibody coupled with fluorescein was added and incubated at 25 °C for 1 h. Hoechst was used to localize the nucleus and Dil was used to localize the cell membrane. Finally, fluorescence microscopy was used to observe and obtain images. And immunofluorescence staining of tissues was performed according to the previously reported method, which increased the section preparation process compared to cell staining.

### PBMCs Isolation and Culture

Human blood and tumor samples were collected at the Department of Hepatobiliary Surgery of Wuhan Union Hospital during surgery for CCA without additional manipulation of the patient and without damaging the integrity of the pathological specimen. All patients sign an informed consent form prior to surgery. All processes complied with the guidelines set by the Ethics Committee of Uinon Hospital, Tongji Medical College, HUST, Wuhan, China (UHCT230220) and followed the Helsinki.

Human blood was collected using a container containing heparin and transferred to the laboratory in an ice bath. This was followed immediately by gradient centrifugation using human peripheral blood lymphocyte isolate and collection of white layer cells, which were PBMCs. Induced T cells were obtained by anti‐CD3 (5 µg mL^−1^) and anti‐CD28 (2 µg mL^−1^) stimulation of PBMCs for 3 days. Whereas stimulation of PBMCs with GM‐CSF and IL‐6 (each 10 ng mL^−1^) for 7 days produced MDSCs. Naive DC cells were then induced by GM‐CSF and IL‐4 for 7 days to generate. Both PBMCs and induced immune cells were cultured using RPMI‐1640 with an appropriate increase in FBS (≥10%) content.

To verify the activation of the ICD effect induced by drug‐laden liposome treatment, treated QBC‐939 cells were co‐cultured with induced naïve DC cells in vitro. And the recovered cells were collected after 24 h and the ratio of CD11C^+^CD80^+^CD86^+^ cells was detected using flow cytometry.

A similar in vitro co‐culture system was used to continue to validate the T cell‐mediated antitumor immune response to treated QBC‐939. Here, CFSE was used to pre‐label the induced T cells. After recounting, QBC‐939 cells were planted back into the original treatment medium and CFSE‐labeled T cells were added proportionally. And IL2 (200 IU mL^−1^) was added to the co‐culture system to stimulate T cell proliferation. T cell proliferation rate was calculated based on the decay of CFSE fluorescence intensity. The in vitro MDSCs functional transformation assay was similar to the above method.

### Distribution Imaging In Vivo

In vivo CMArg@Lip distribution imaging was detected in tumor‐bearing Balb/c nude mice. After subcutaneous injection of 5 × 10^6^ QBC‐939 cells, CCA with a volume of ≈300 mm^3^ grew in the hind legs of tumor‐bearing Balb/c nude mice in ≈2 weeks. After 150 uL of CMArg@Lip (20 µg mL^−1^) was injected into tumor‐bearing Balb/c nude mice via tail vein, the in vivo spectral images under 650 nm excitation light were examined in real time using In‐Vivo FX PRO. And the in vivo organ distribution of CMArg@Lip was detected in mice organs harvested at 12 and 24 h after drug injection.

### Cholangiocarcinoma Homologous Graft Model

To clarify the powerful immunomodulatory role of CMArg@Lip, a homologous transplant tumor model to establish a CCA model subcutaneously in C57BL/6 mice was used. This model preserves the intact immune system of the mice while avoiding the damage to the mice caused by repeated opening of the abdominal cavity during treatment in the primary tumor. By tail vein hyperbaric transfection, transposase and plasmids carrying AKT and Notch internal domain transposons were injected into C57BL/6. And the mice quickly developed primary CCA at ≈1 month. The CCA tissue was collected after its execution and removed as much normal tissue as possible was removed and subsequently cut into 1–2 mm^3^ and implanted under the subcutis of another C57BL/6 under Matrigel coating. About 1 month later, tumor‐bearing mice with uniformly sized transplanted tumors (≈200 mm^3^) were selected, injected with different drug‐loaded liposomes in the tail vein and the tumor area was treated with light (650 nm, 100 mW cm^−2^) for 15 min after 12 h. Tumor volumes (*n* = 5) were measured every 3 days in treated mice and survival (*n* = 7) was recorded. Mice were also considered dead when their tumor volumes were greater than 2000 mm^3^. And all surviving mice were executed on the 90th day after treatment. In addition, 6 mice receiving different treatments were executed 1 week after treatment and the treated tumors were collected for IHC and immunofluorescence staining. Six mice were also executed for flow cytometry assays to determine the altered tumor immune microenvironment after different liposome treatments.

### Biosecurity Detection

In vitro safety was assessed using a previously reported method using high doses of CMArg@Lip in incubated mouse blood and observed for hemolysis. In vivo systemic toxicity was revealed by monitoring body weight changes in mice during treatment, on the one hand, and by obtaining organs for HE staining and blood for cell counts and biochemical parameters after increasing the drug dose, on the other hand.

### Statistical Analysis

All results were presented in the form of mean ± standard deviation (SD) and underwent statistical analysis using GraphPad Prism 8 software. Statistical disparities between the two groups were determined through a one‐way analysis of variance (ANOVA) with a subsequent Tukey test, and significance was established at a *p*‐value of less than 0.05. The notation of an asterisk (^*^) indicates statistical significance observed between the respective bars (ns, no statistical difference; ^*^, *p* < 0.05; ^**^, *p* < 0.01; ^***^, *p* < 0.001).

## Conflict of Interest

The authors declare no conflict of interest.

## Author Contributions

W.W., Y.G., and J.X. contributed equally to this work and shared the first author. Q.Z. and X.C. are both co‐last authors. Y.G. designed and characterized the drug‐loaded liposomes. W.W. and J. X. performed the cell and animal experiments. W.W. and Y.G. analyzed the data and wrote the manuscript. T.Z., B.Y., and S.H. participated in the design of the experimental protocol and completed some of the experiments. Q.Z., Y.X., and X.C. supervised the research and revised the manuscript. All authors reviewed the manuscript.

## Supporting information

Supporting Information

## Data Availability

The data that support the findings of this study are available from the corresponding author upon reasonable request.
