# Supplementary material for: A NRF2 Regulated and the Immunosuppressive Microenvironment Reversed Nanoplatform for Cholangiocarcinoma Photodynamic‐Gas Therapy
Source: Adv Sci (Weinh). 2024 Feb 2;11(14):2307143. doi: 10.1002/advs.202307143 (PMC11005733; doi:10.1002/advs.202307143)
Supplement: Supplementary file 1 — Supporting Information [file ADVS-11-2307143-s001.pdf]

ADVANCED SCIENCE
Open Access

Supporting Information

for *Adv. Sci.*, DOI 10.1002/advs.202307143

A NRF2 Regulated and the Immunosuppressive Microenvironment Reversed Nanoplatform for Cholangiocarcinoma Photodynamic-Gas Therapy

Weimin Wang, Yang Gao, Jianjun Xu, Tianhao Zou, Bin Yang, Shaobo Hu, Xiang Cheng, Yun Xia* and Qichang Zheng**

Supporting Information

A NRF2 Regulated and the Immunosuppressive Microenvironment Reversed Nanoplatform for Cholangiocarcinoma Photodynamic-Gas Therapy

Weimin Wang, Yang Gao, Jianjun Xu, Tianhao Zou, Bin Yang, Shaobo Hu, Xiang Cheng, Yun Xia*, Qichang Zheng**

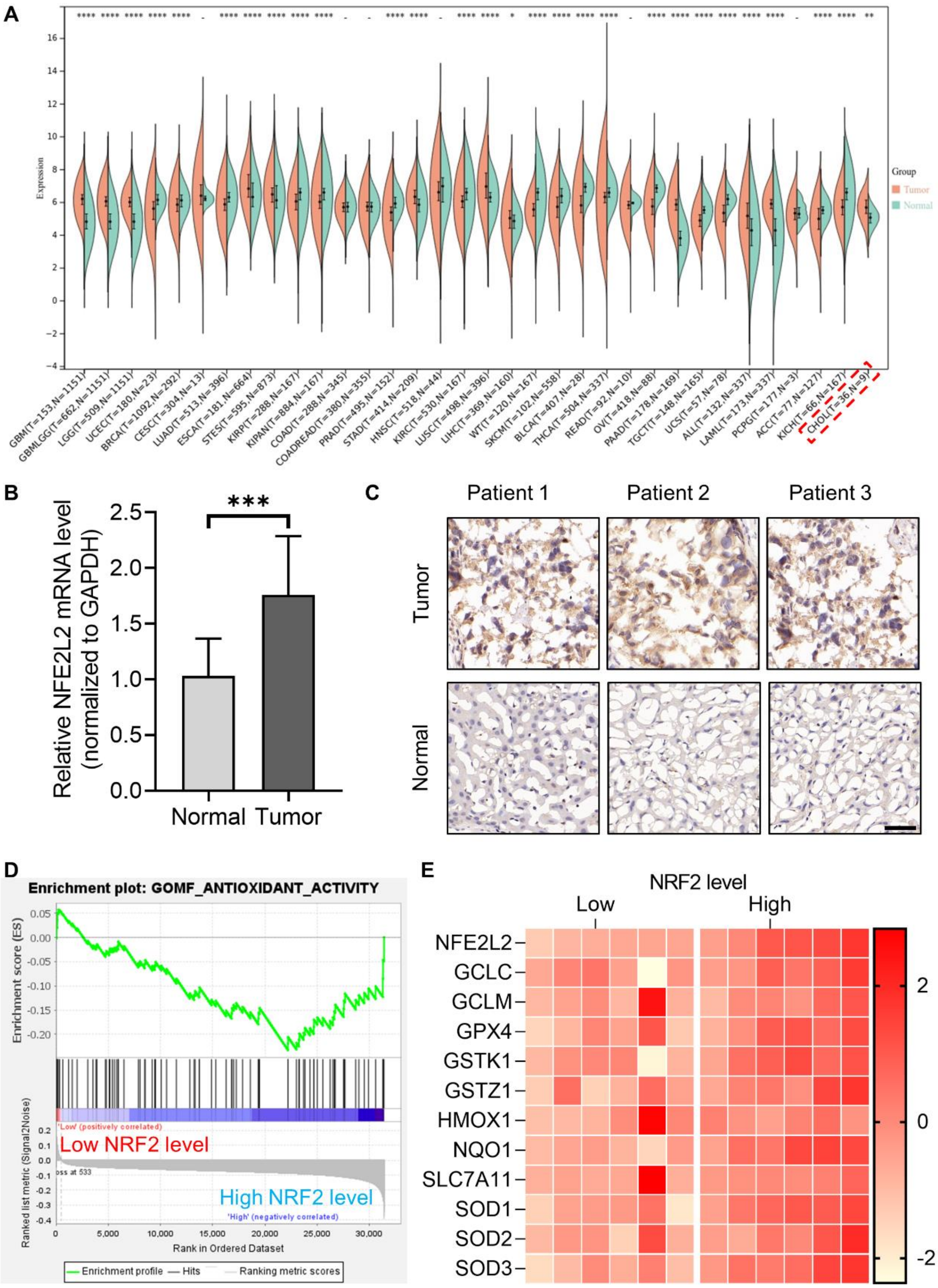

Figure S1. Expression of antioxidant genes in CCA. **(A)** Pan cancer analysis of NRF2 expression by TCGA database. **(B)** NRF2 mRNA levels in cancer and paracancer tissues (n=15). **(C)** IHC images of NRF2 in cancer and paracancerous tissues. Scale bar: 50 μm. (D) GSEA enrichment analysis of antioxidant gene sets between low and high NRF2 level by

GSE84756 data set. (E) Heat map of representative antioxidant gene expression after grouping by the median expression level of NRF2 mRNA.

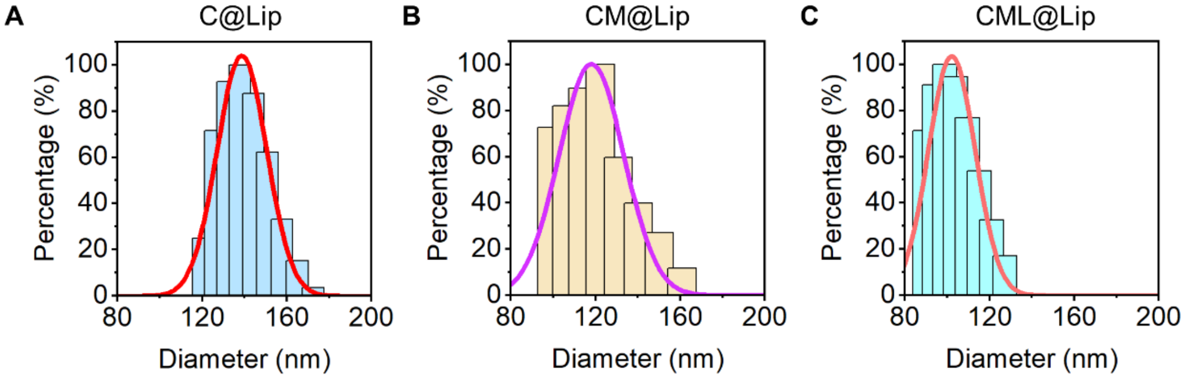

Figure S2. Particle size distribution of liposomes. **(A)** C@Lip. **(B)** CM@Lip. **(C)** CMArg@Lip.

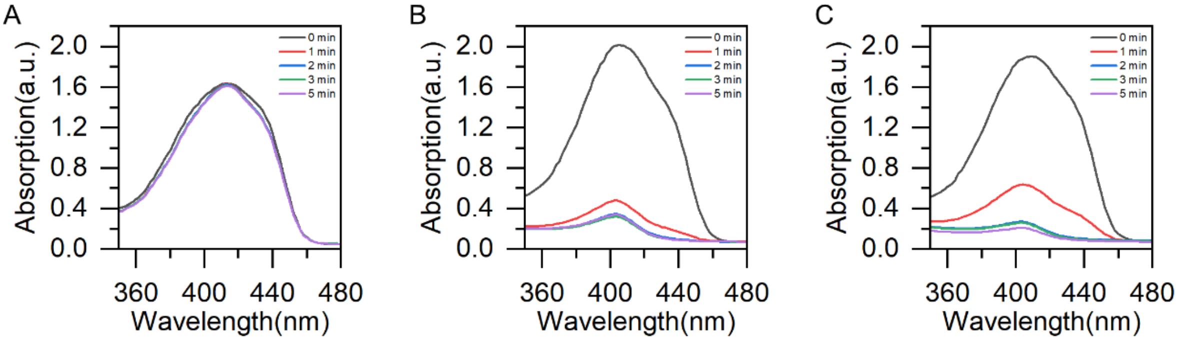

Figure S3. ROS generation of Ce6 in different liposomes. **(A)** Blank. **(B)** C@Lip. **(C)** CM@Lip.

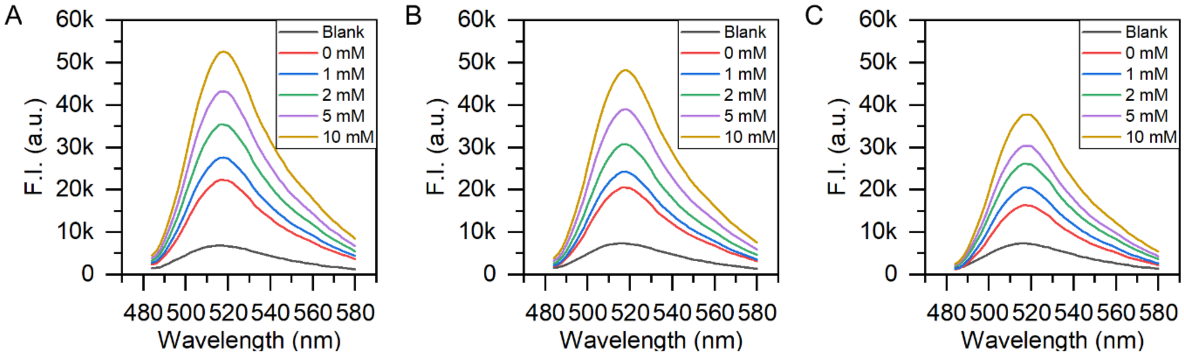

Figure S4. NO generation of Arg@Lip in an environment with different concentrations of H_2O_2 and different pH. (A) pH 5.5, (B) pH 6.8, (C) pH 7.4.

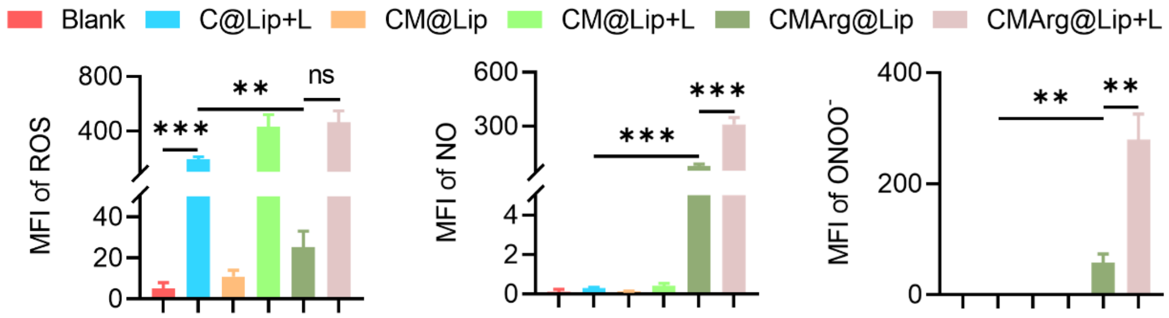

Figure S5. The mean fluorescence intensity of the fluorescence images in Figure 2C.

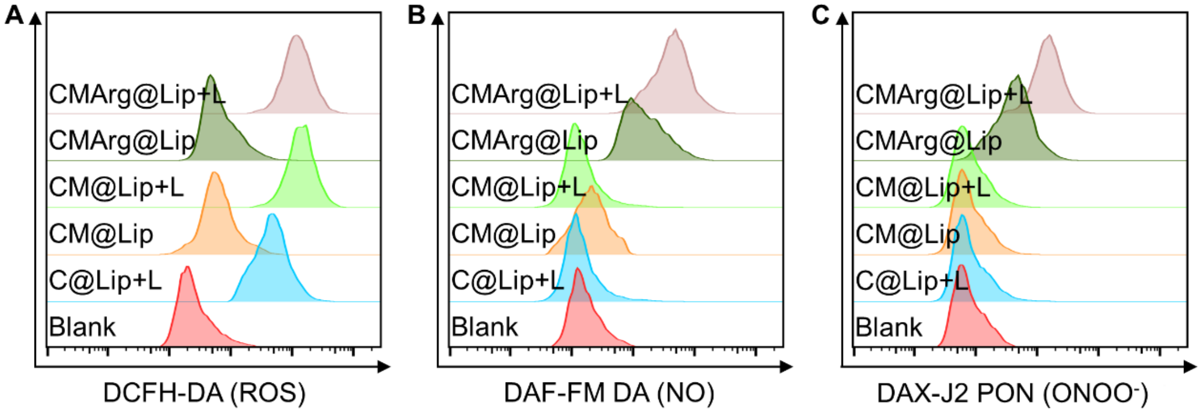

Figure S6. Flow cytometry analysis of the production of ROS, NO and $ONOO^-$ in various groups. **(A)** ROS was detected by DCFH-DA after different treatments. **(B)** NO was detected by DAF-FM DA after different treatments. **(C)** $ONOO^-$ was detected by DAX-J2 PON Green after different treatments.

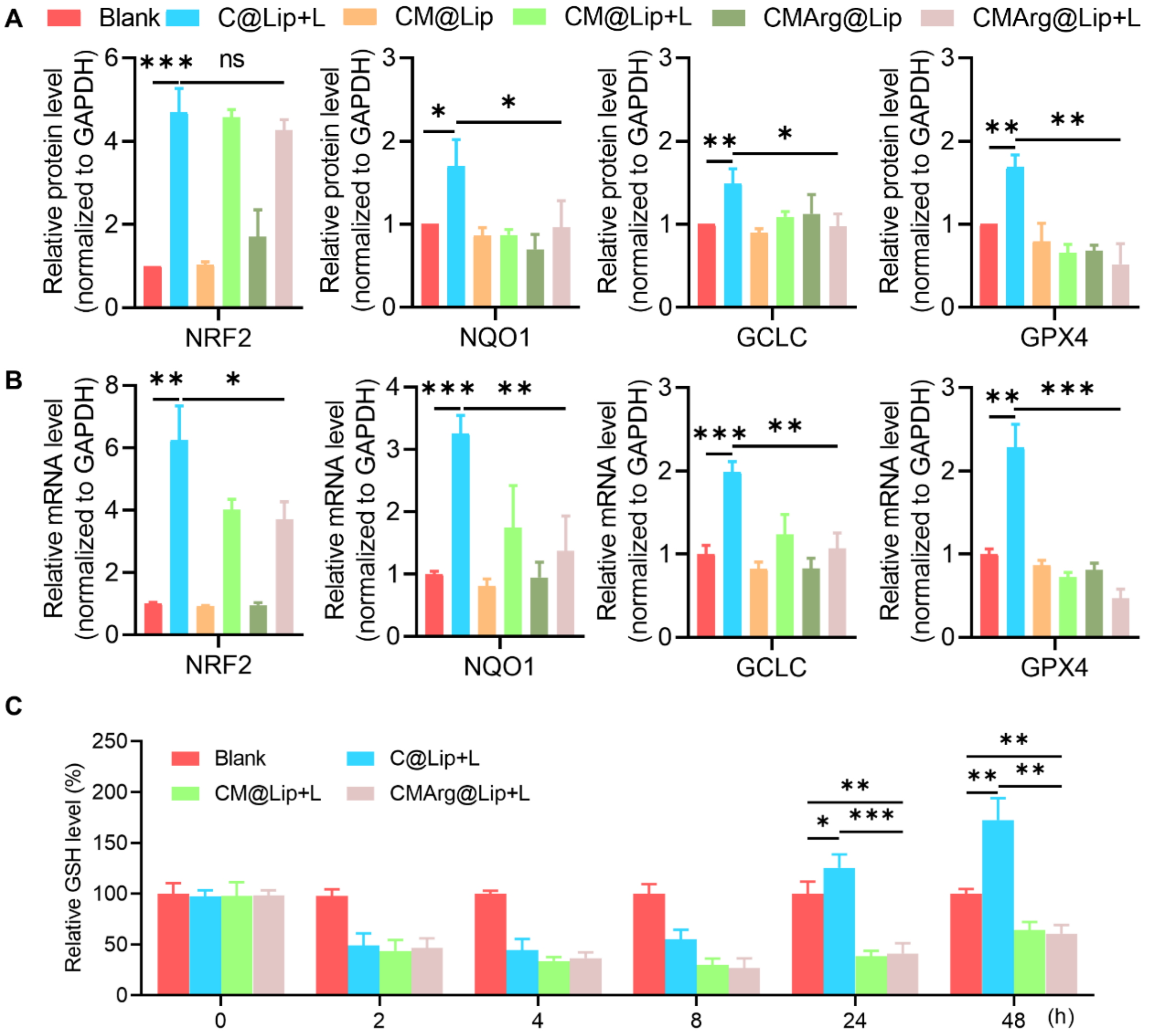

Figure S7. Detection of intracellular antioxidant system after different treatments. **(A)** and **(B)** WB and qPCR analysis of NRF2, NQO1, GCLC and GPX4. **(C)** The relative level of intracellular GSH was detected at various times after different treatments.

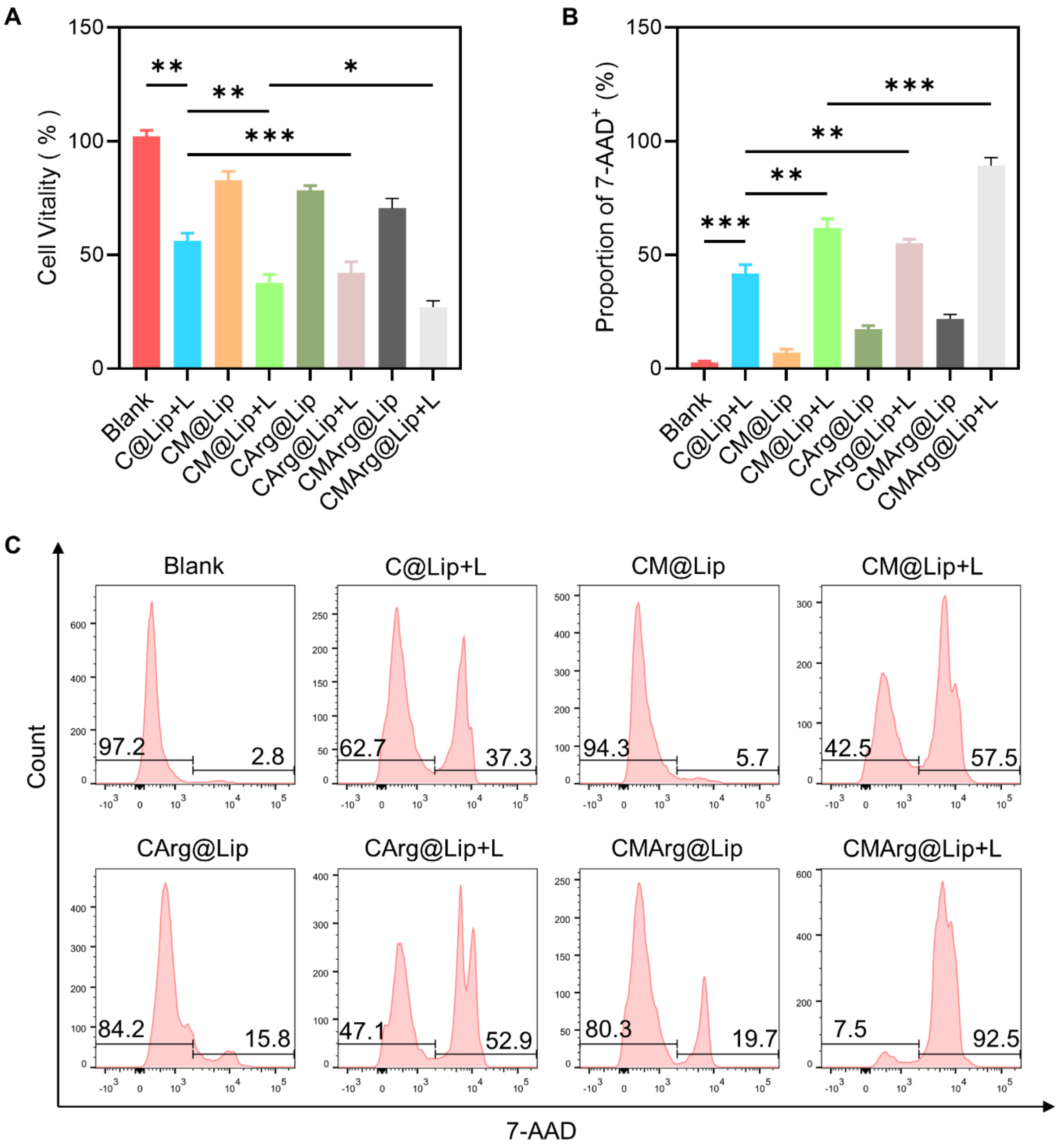

Figure S8. Cell viability and cell death after different treatments. **(A)** Detection of cell vitality after different liposomes (dose: 20 μg/ml Ce6) with or without light (650 nm, 100 mW/cm^2, 15 min) by CCK8. **(B)** and (**C)** Flow cytometry typical images and statistical analysis of cell death (7-AAD$^+$) under different conditions.

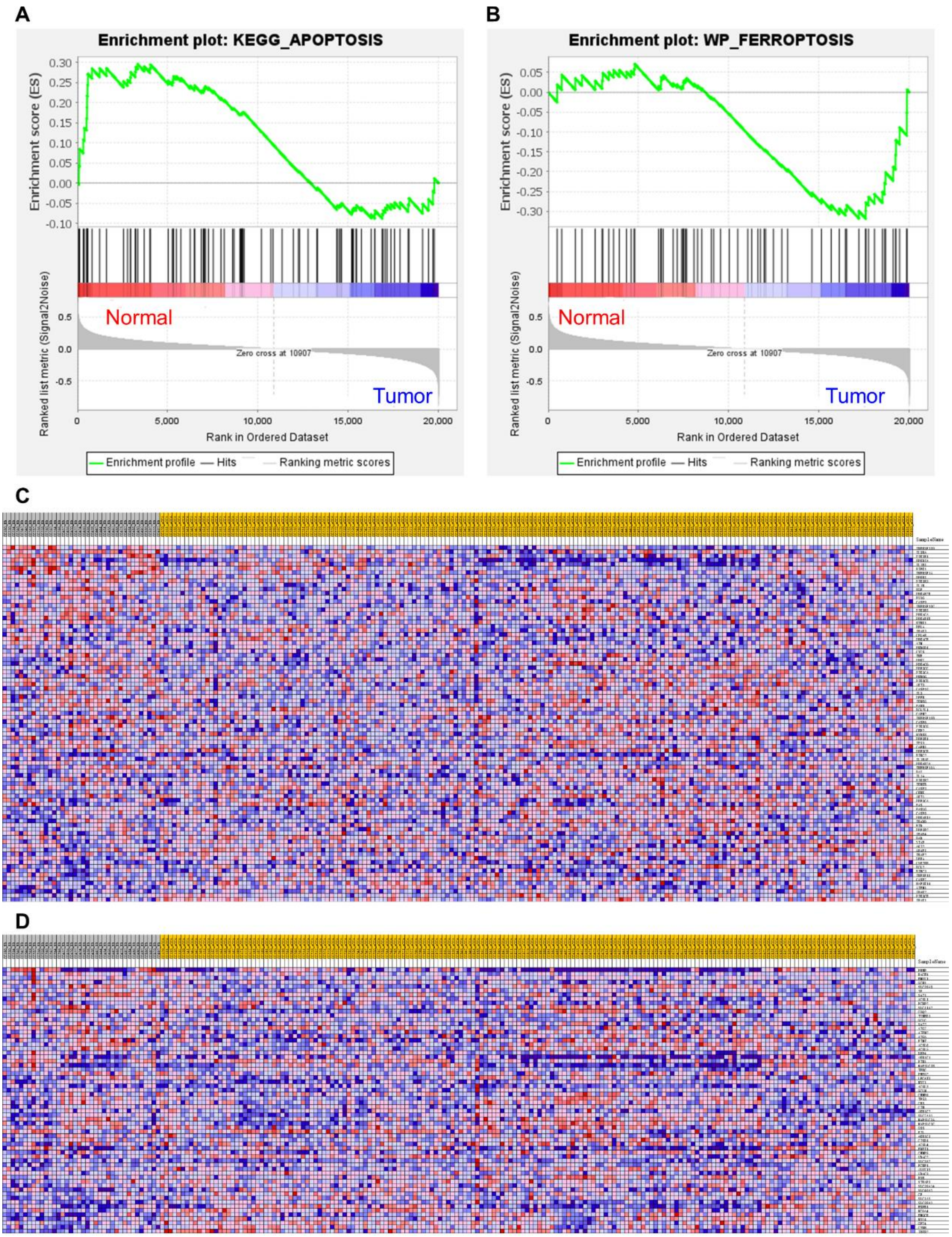

Figure S9. GSEA analysis of the GSE132305 dataset. (**A)** GSEA enrichment analysis of apoptosis gene sets between tumor and normal tissues. (**B)** GSEA enrichment analysis of ferroptosis gene sets between tumor and normal tissues. (**C)** Heat map of apoptosis genes (normal vs tumor). (**D)** Heat map of ferroptosis genes (normal vs tumor).

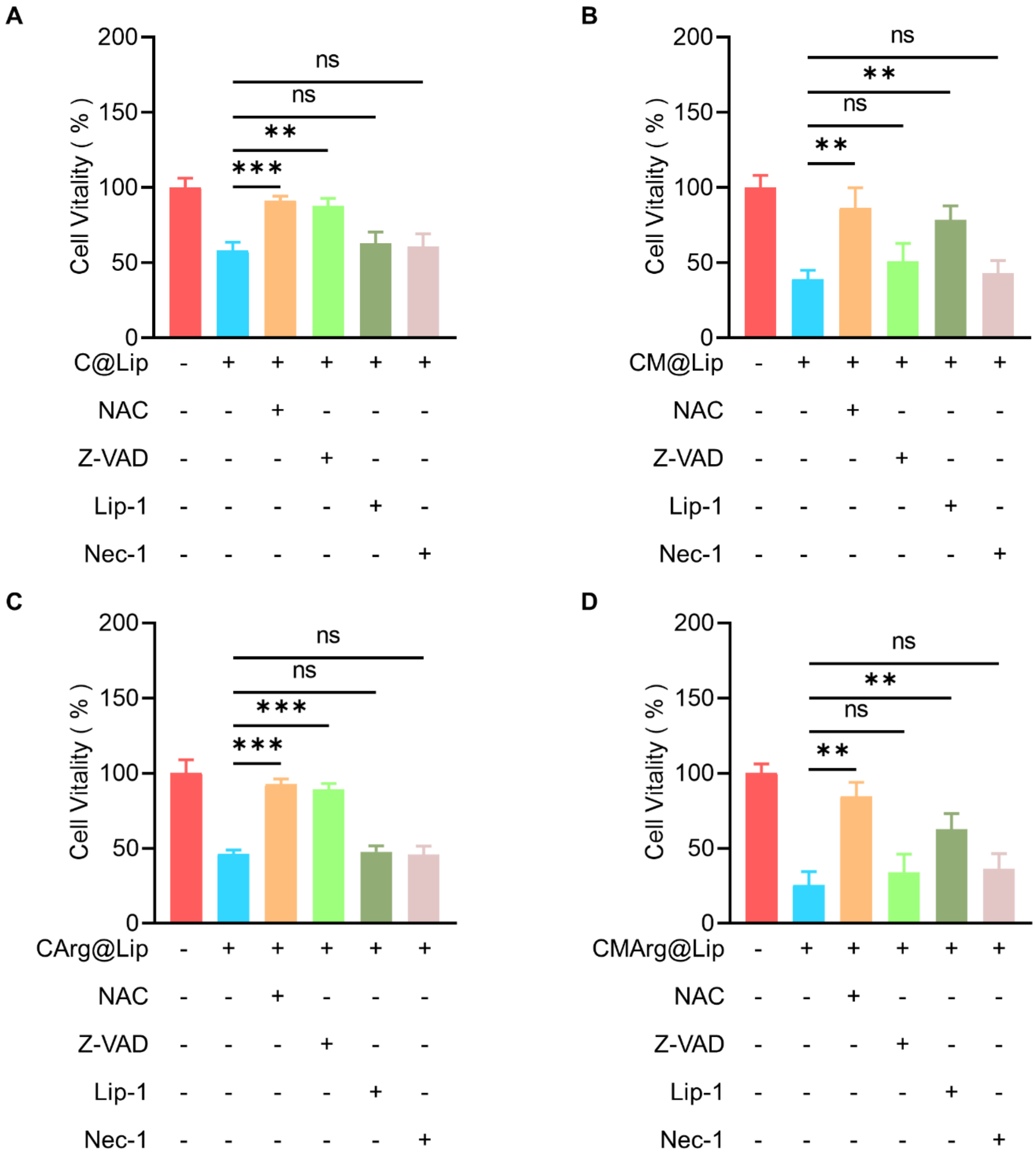

Figure S10. CCK8 assays for cell death modality. Pretreatment of QBC-939 cells with different programmed cell death blockers followed by phototherapy with **(A)** C@Lip **(B)** CM@Lip **(C)** CArg@Lip **(D)** CMArg@Lip and detection of cell viability changes using CCK8. NAC: N-Acetylcysteine, Z-VAD: Z-VAD(OMe)-FMK, Lip-1: Liproxstatin-1, Nec-1: Necrostatin-1.

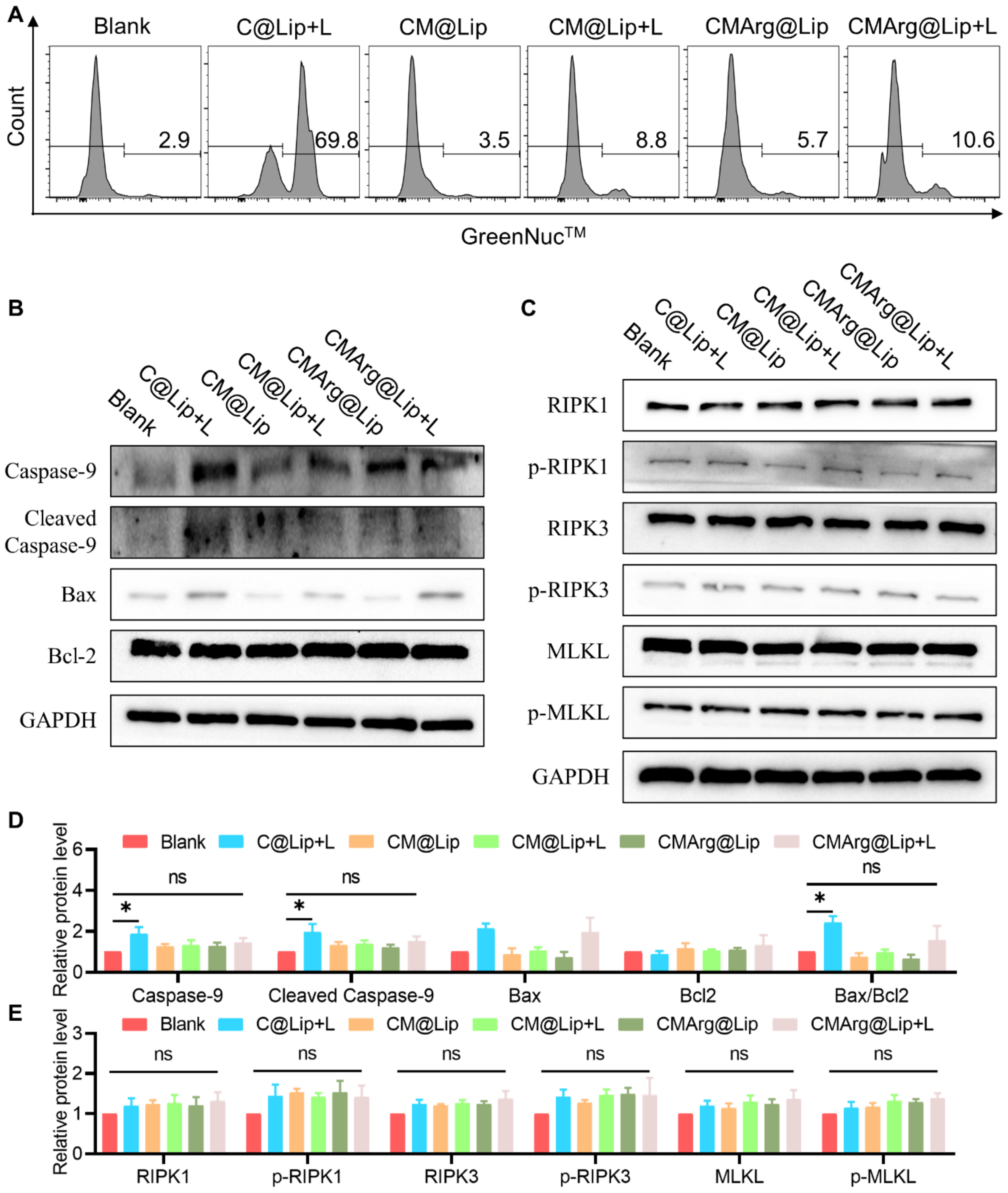

Figure S11. Flow cytometry and WB assays for cell death modality. **(A)** Detection of caspase-3 activation by flow cytometry. **(B)** and **(D)** WB detection of apoptosis pathway proteins (caspase-9, cleaved caspase-9, Bax and Bcl-2) in QBC-939 receiving different treatments, and statistical analysis of grayscale values. **(C)** and **(E)** WB detection of necroptosis pathway proteins (RIPK1, p-RIPK1, RIPK3, p-RIPK3, MLKL and p-MLKL) in QBC-939 receiving different treatments, and statistical analysis of grayscale values.

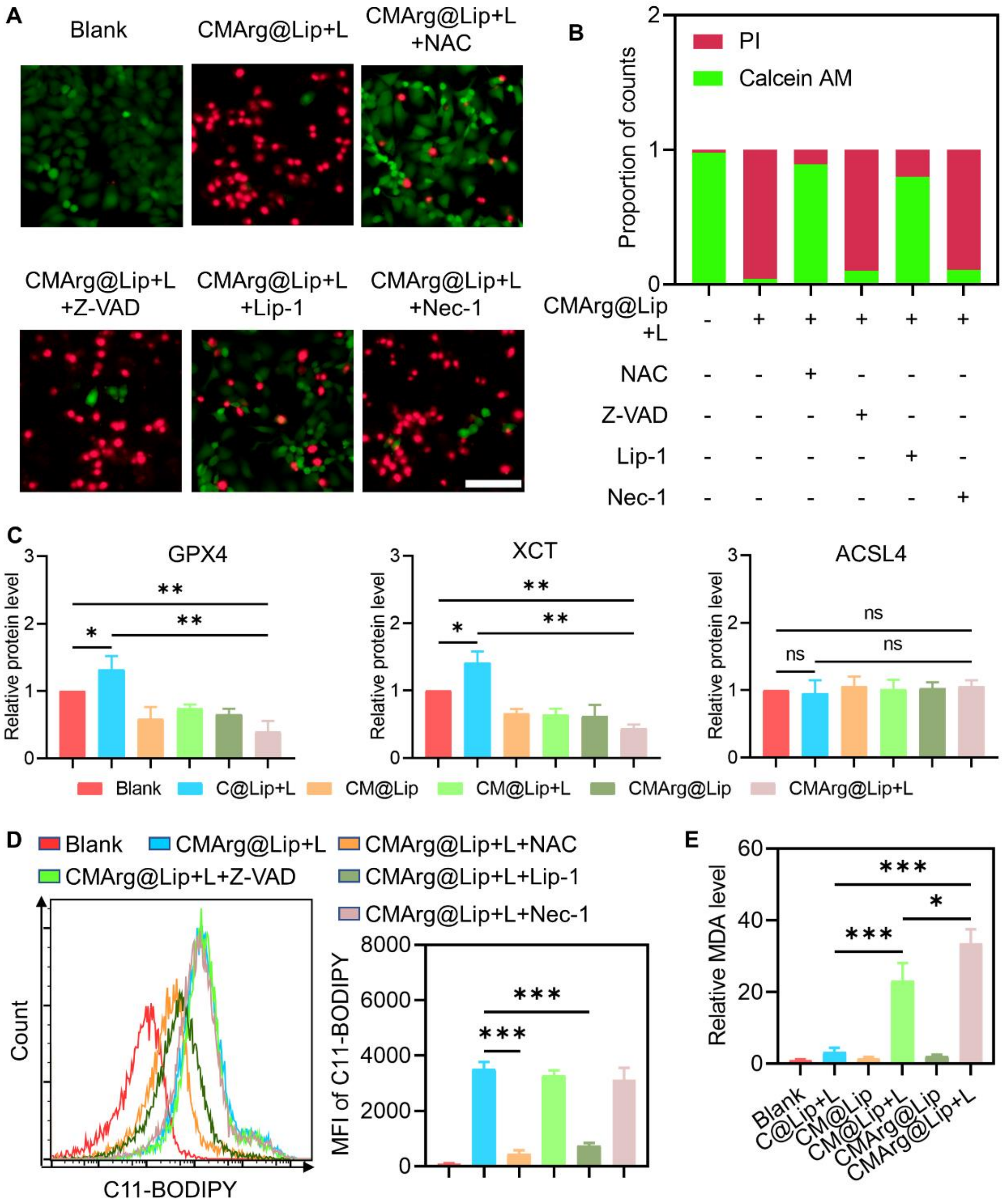

Figure S12. Detection of ferroptosis induced by CMArg@Lip with light treatment. **(A)** Calcein-AM/PI staining for detection of CMArg@Lip+L treatment preincubated with different PCD inhibitors in QBC-939. Scale bar: 100 μm. **(B)** Statistical analysis of Calcein-AM/PI staining. **(C)** Statistical analysis of GPX4, XCT and ACSL4 protein levels by WB detection after different treatments. **(D)** Flow cytometry analysis of lipid peroxidation in QBC-939 cells after different treatments by C11-BODIPY. **(E)** Detection of MDA levels in QBC-939 after different treatments.

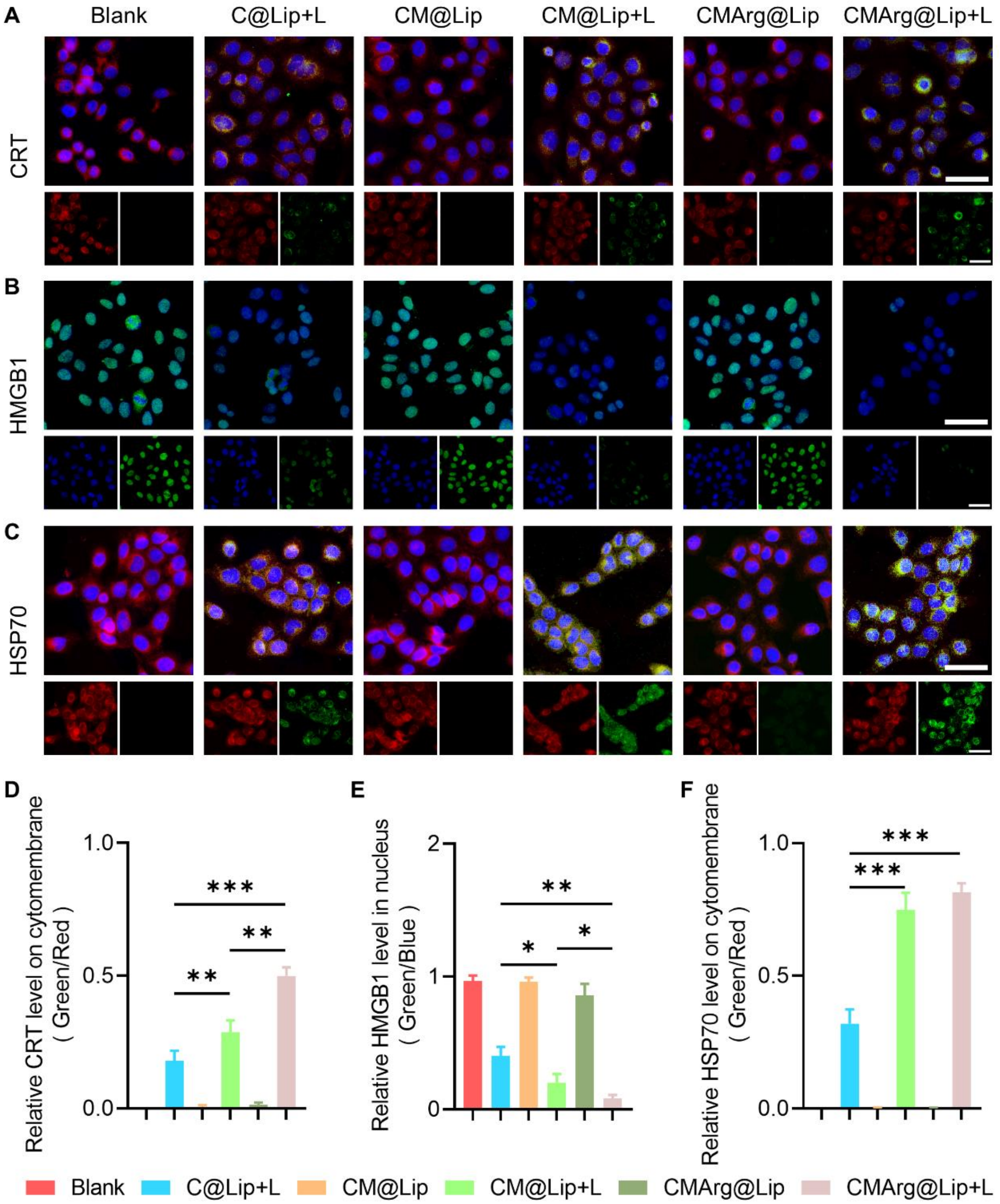

Figure S13. Immunofluorescence detection of ICD effects after treatment with different liposomes. **(A)** Immunofluorescence images of CRT after different treatments. (Green: CRT, Red: cytomembrane, Blue: nucleus, Scale bar: 50 μm). **(B)** Immunofluorescence images of HMGB1 after different treatments. (Green: HMGB, Blue: nucleus, Scale bar: 50 μm). **(C)** Immunofluorescence images of HSP70 after different treatments. (Green: HSP70, Red: cytomembrane, Blue: nucleus, Scale bar: 50 μm). **(D)**, **(E)** and **(F)** Statistical analysis of CRT, HMGB1 and HSP70 immunofluorescence images.

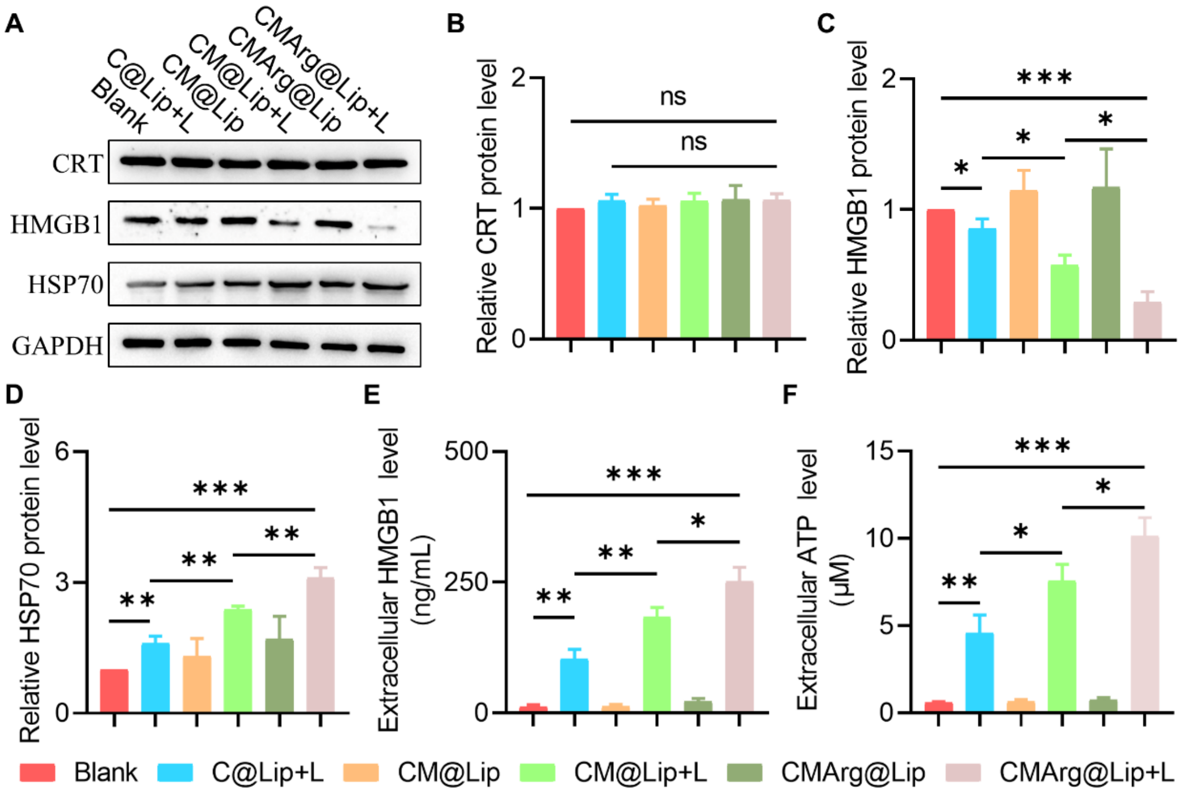

Figure S14. WB and Elisa detection of ICD effects after different treatments. **(A)** WB analysis of CRT, HMGB1 and HSP70 in QBC-939 after different treatments. **(B)**, **(C)** and **(D)** Statistical analysis of WB detection CRT, HMGB1 and HSP70. **(E)** and **(F)** Elisa assays for extracellular HMGB1 and ATP.

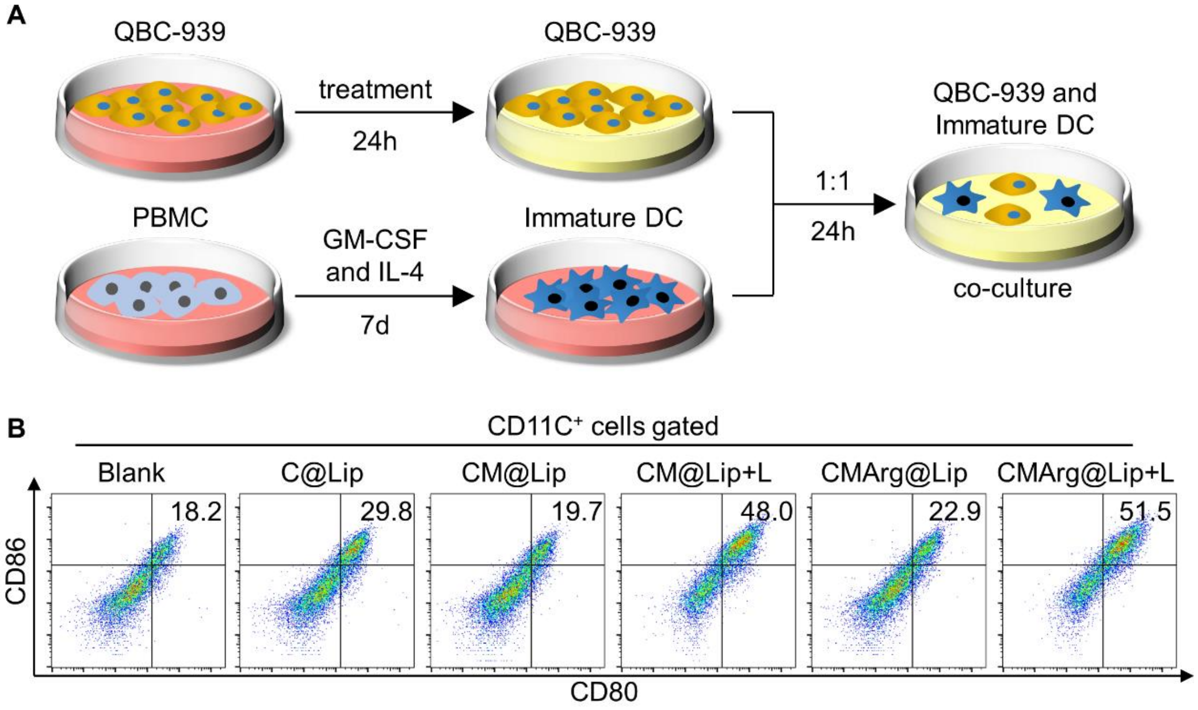

Figure S15. Detection of DC maturation in vitro. **(A)** Schematic diagram of co-culture in vitro of immature DC and QBC-939 cells. **(B)** Flow cytometry detection of mature DC ($CD11C^+CD80^+CD86^+$) cells in co-culture system.

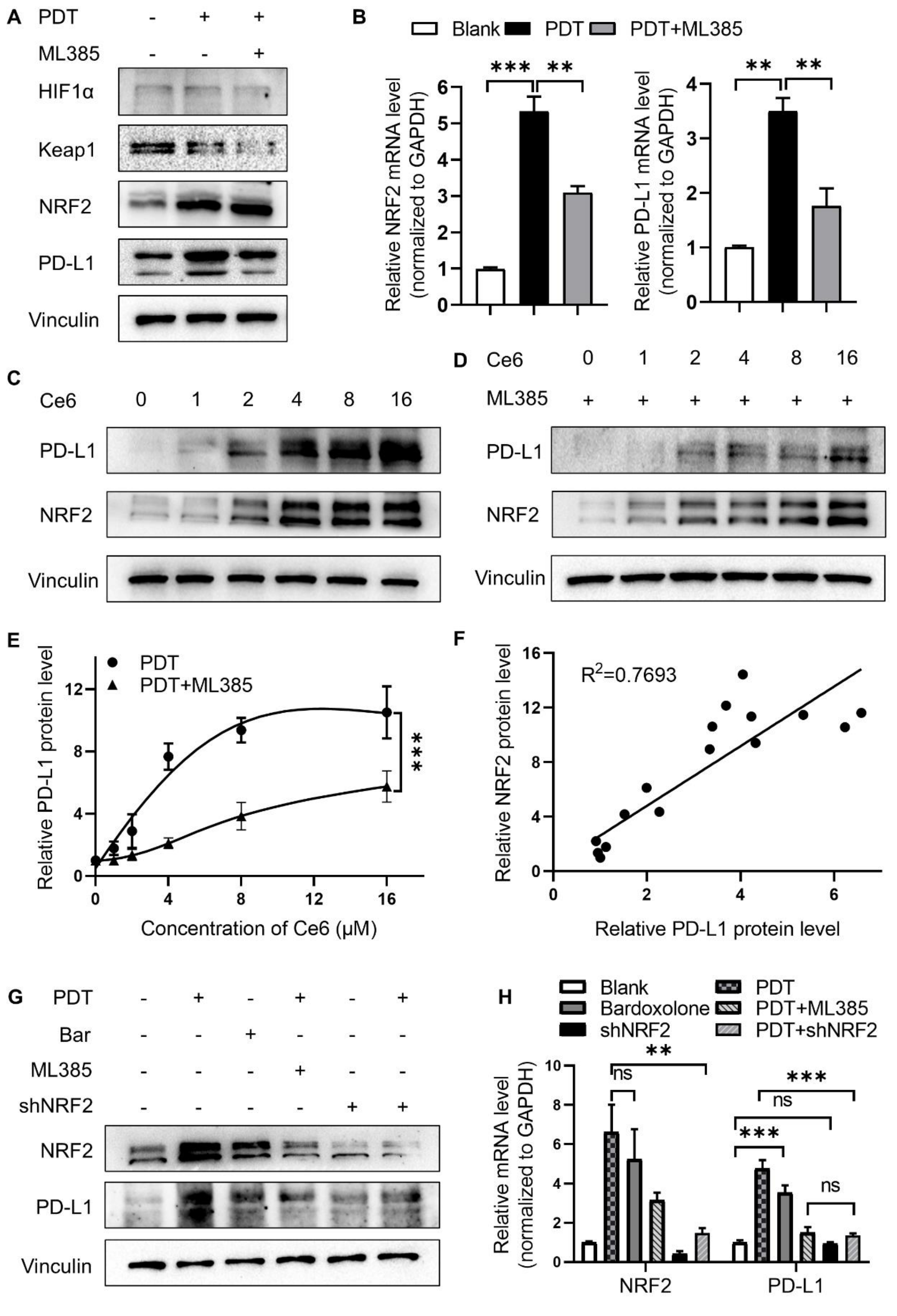

Figure S16. PDT up regulates PD-L1 expression through NRF2. (**A)** WB analysis of HIF1α, NRF2 and PD-L1 after PDT or combined with ML385 treatment in oxygen enriched

environment. (**B)** qPCR analysis of NRF2 and PD-L1 after PDT or combined with ML385 treatment. (**C)** and (**D)** WB analysis of NRF2 and PD-L1 after different concentrations of Ce6 or combined with ML385 treatment. Unit: μM. (**E)** Gray value analysis of PD-L1 in C and D by Image J. (**F)** Correlation analysis of gray value between NRF2 and PD-L1 in C. (**G)** and (**H)** WB or qPCR analysis of NRF2 and PD-L1 after various treatments. (Ce6: 5μM. Light irradiation: 650 nm, 100mW/cm^2, 15 min. ML385: 5μM. Bardoxolone: 5μM)

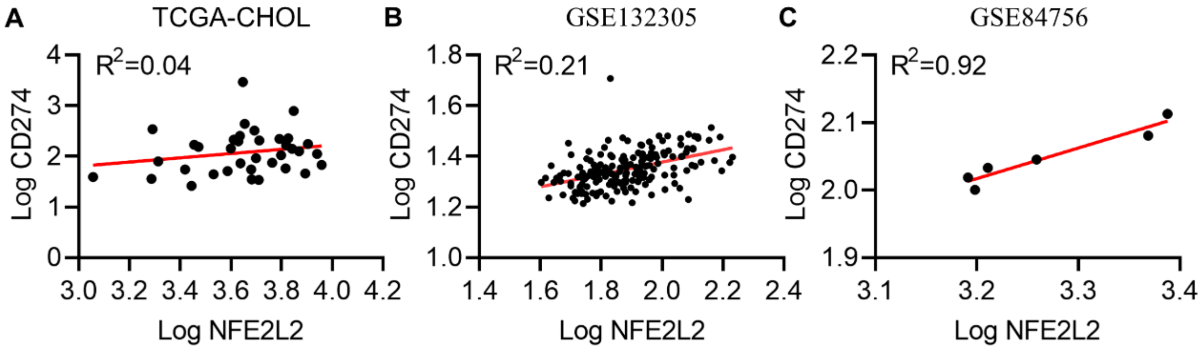

Figure S17. Correlation analysis between NRF2 and PD-L1. **(A)** Analysis by TCGA-CHOL data set. **(B)** Analysis by GSE132305 data set. **(C)** Analysis by GSE84756 data set.

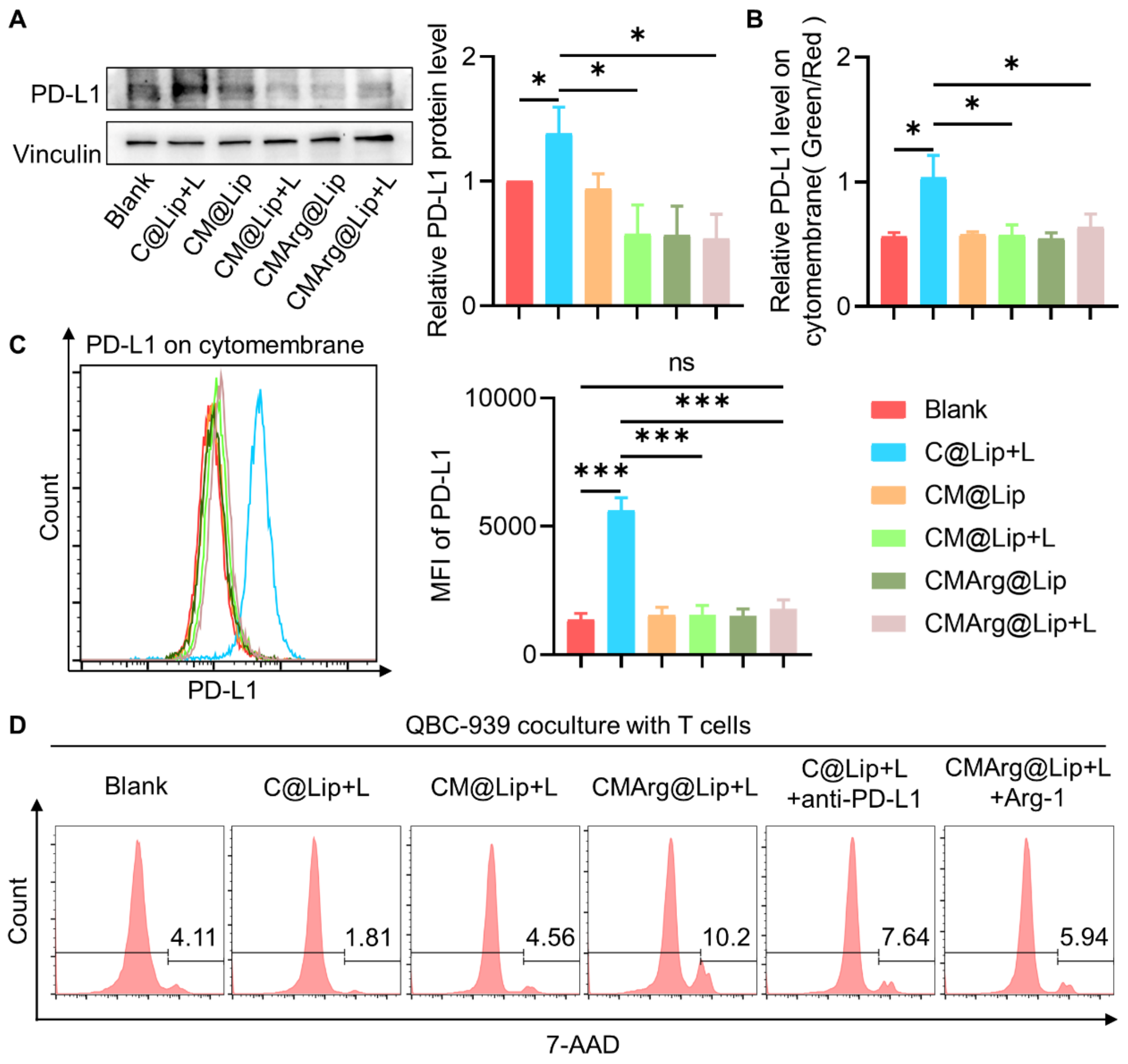

Figure S18. CMArg@Lip effectively reversed the upregulation of PD-L1. **(A)** WB analysis of PD-L1 in QBC-939 cells after receiving different treatments. (The right side shows the results of the statistical analysis of grayscale values). **(B)** Analysis of the relative expression of PD-L1 on the cell membrane in Figure 4A. **(C)** Flow cytometry analysis of PD-L1 on QBC-939 cell membranes after receiving different treatments. (The right side shows the statistical analysis of the mean fluorescence intensity). **(D)** 7-AAD assays for QBC-939 cell death after coincubation of QBC-939 after treatment with T cells.

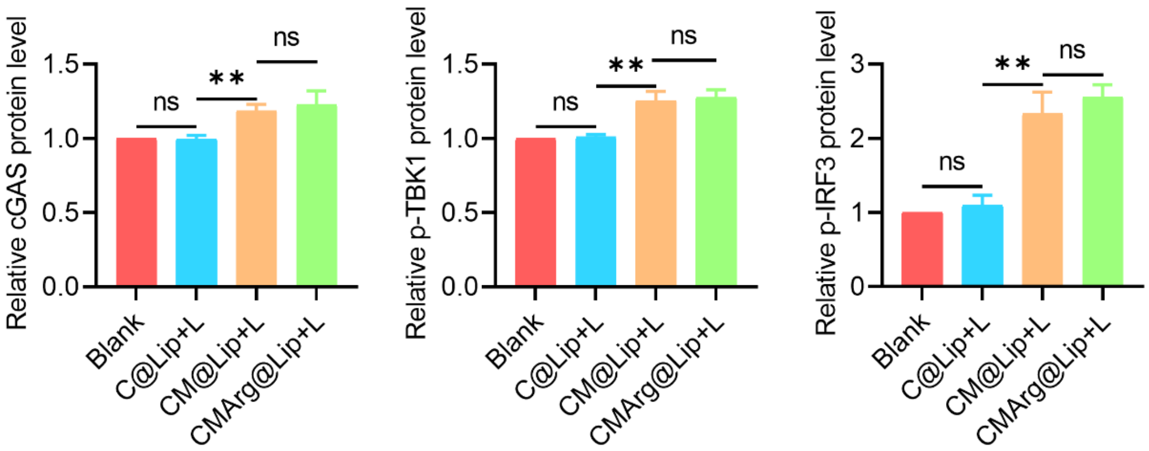

Figure S19. Analysis of gray value after detecting the protein (cGAS, p-TBK1 and p-IRF3) of sting pathway in MDSC by WB.

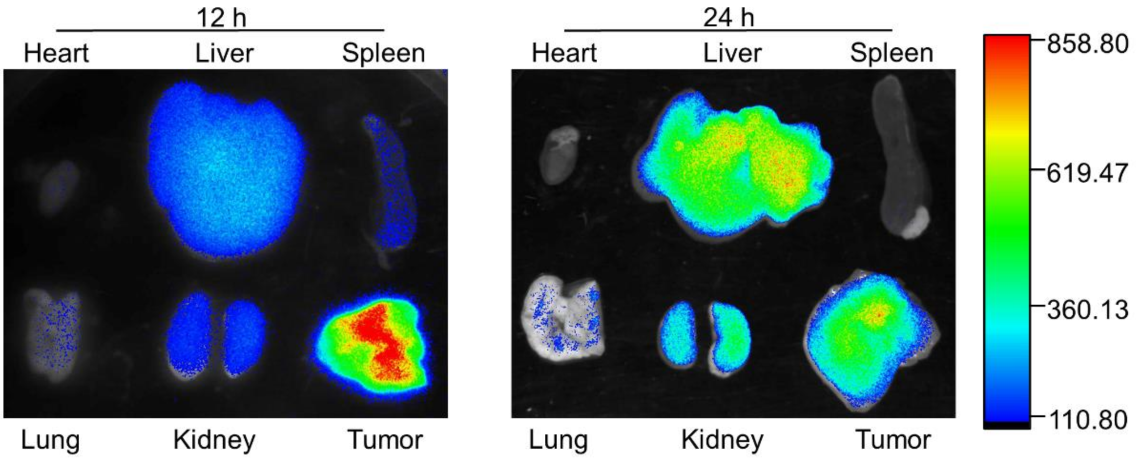

Figure S20. Organ imagings at different time after intravenous injection of CMArg@Lip in nude mice bearing tumor.

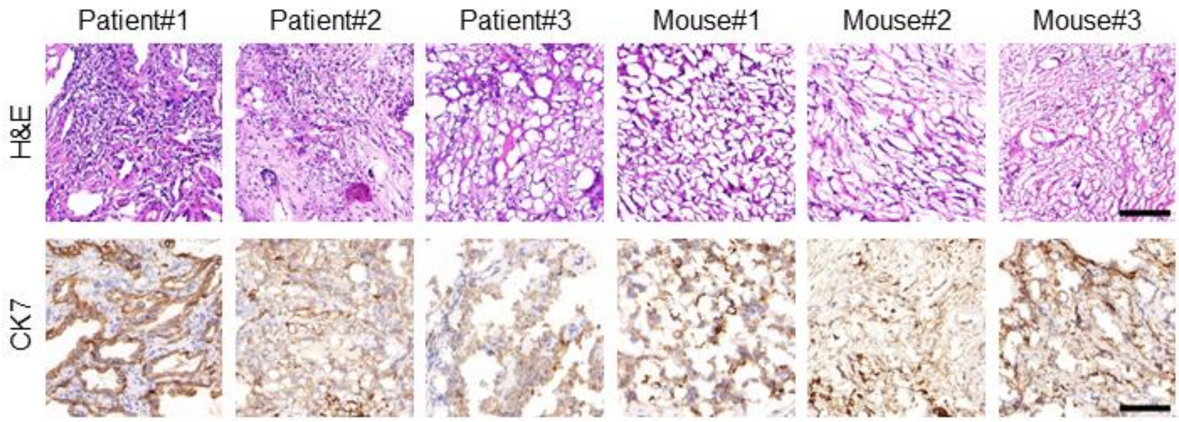

Figure S21. Verification of the model of homograft of CCA. The figures showed the comparison of HE and CK7 staining of specimens from patients with CCA and mouse homologous transplanted tumors. Bar: 100 μm.

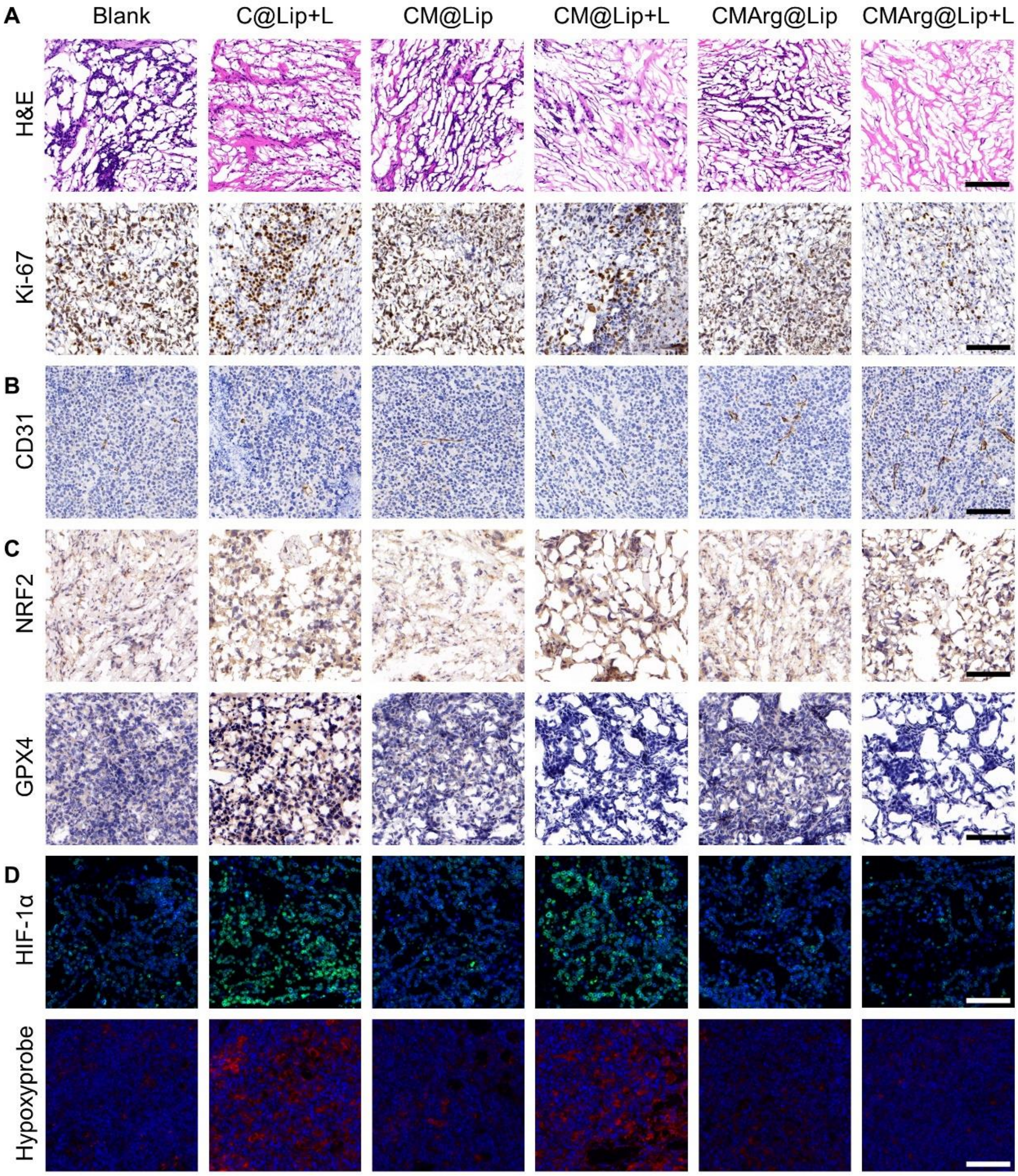

Figure S22. CMArg@Lip inhibited tumor growth in vivo while improving the antioxidant and hypoxic microenvironment. The figure shown immunohistochemistry (H&E, Ki67, CD31, NRF2 and GPX4) and immunofluorescence (HIF-1α and hypoxyprobe) imaging of tumors in mice receiving different treatments. Bar: 100 μm.

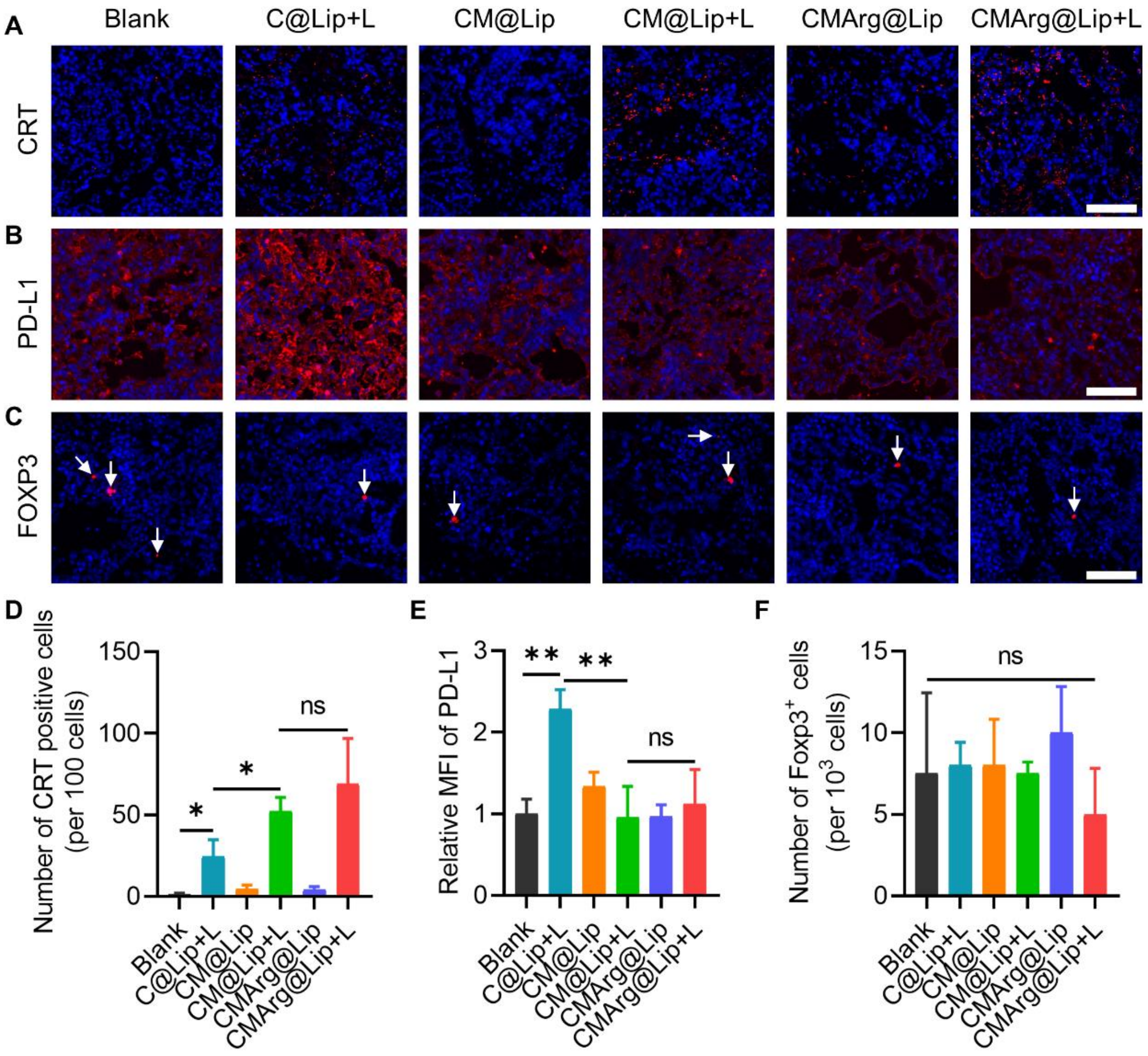

Figure S23. Immunofluorescence staining (A: CRT, B: PD-L1, C: FOXP3, Bar: 100 μm) of tumor tissues of tumor-bearing mice after different treatments and statistical analysis (D: CRT^{+}cells, E: relative mean fluorescence intensity of PD-L1, F: Foxp3^{+} cells).

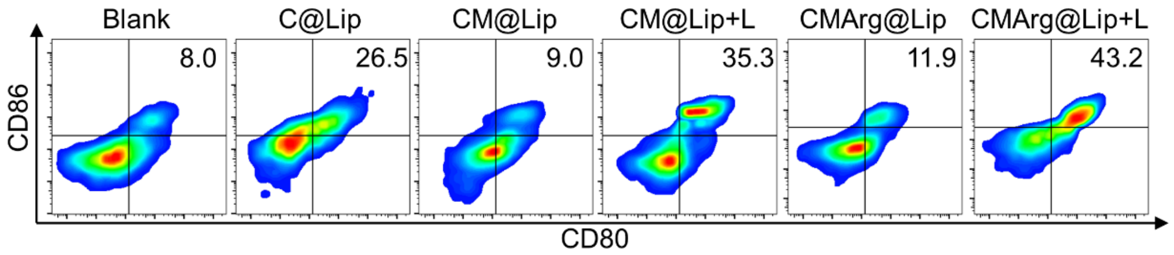

Figure S24. Flow cytometry analysis of the proportion of mature DC cells in tumors of mice receiving different treatments.

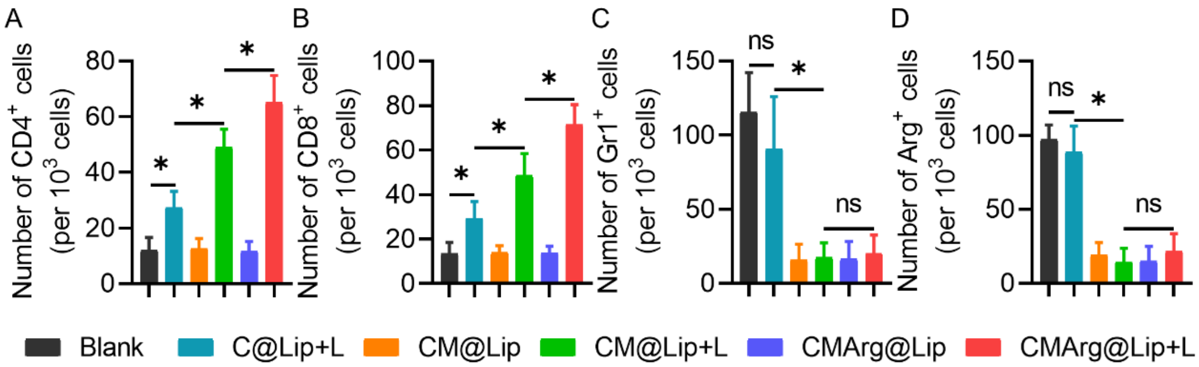

Figure S25. Statistical analysis of immunofluorescence staining images in Figure 6A and B.

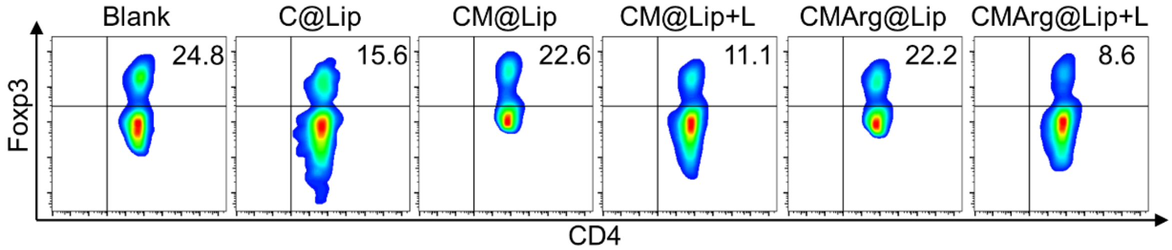

Figure S26. Flow cytometry analysis of Treg cells ($Foxp3^{+}CD4^{+}$) proportions in tumors of mice receiving different treatments.

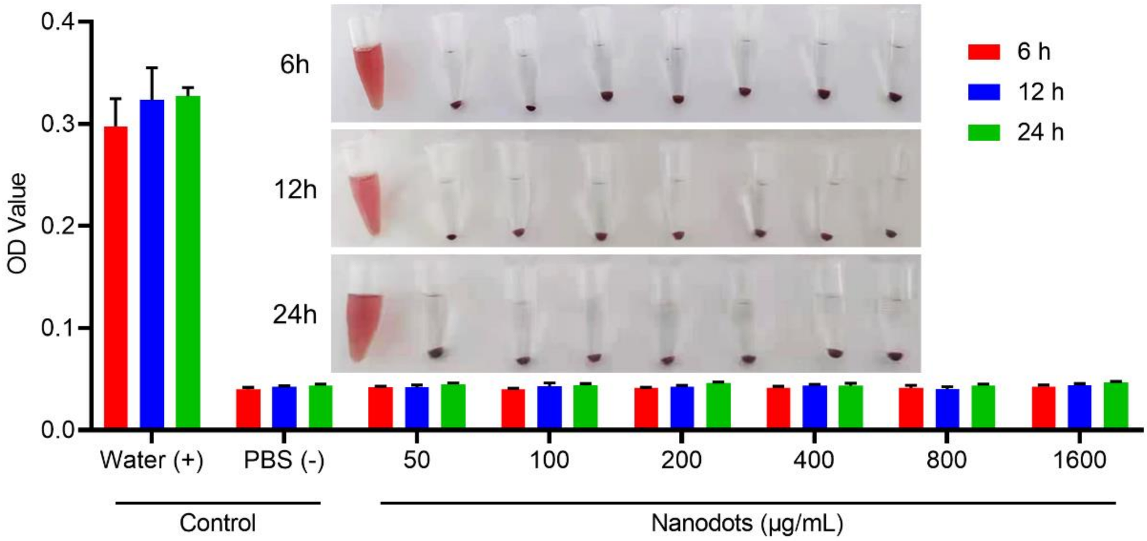

Figure S27. Detection of hemolysis at different time points after in vitro treatment of mouse blood with different doses of CMArg@Lip. (Positive control: water, negative control: PBS)

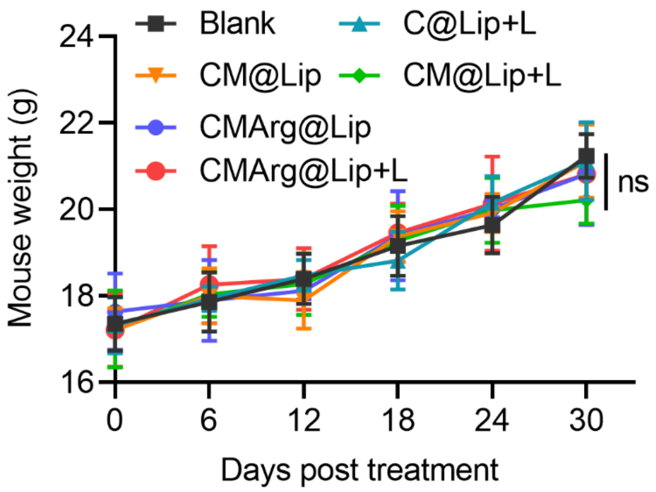

Figure S28. Body weight changes of tumor-bearing mice after different treatments.

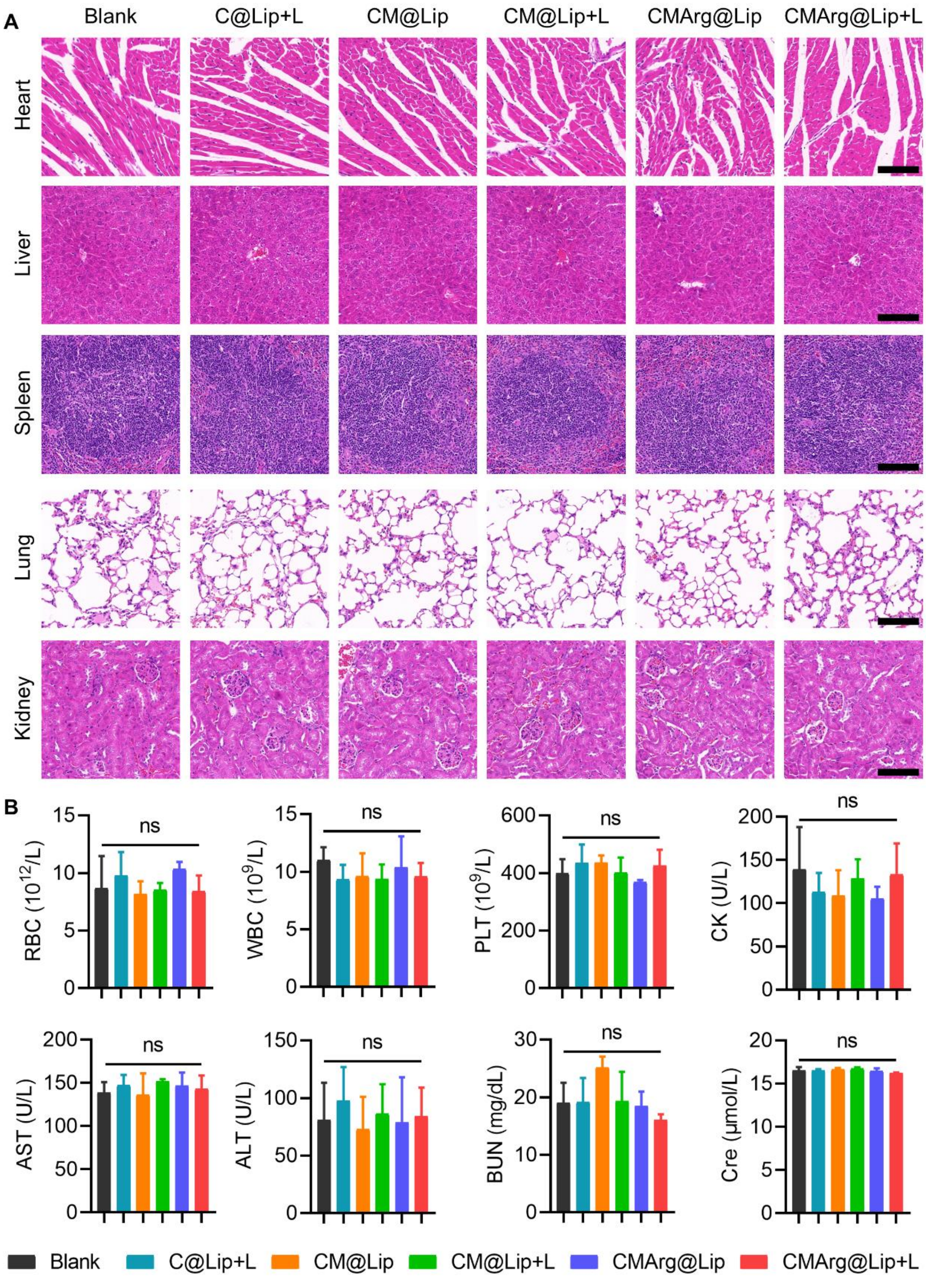

Figure S29. CMArg@Lip had no significant toxic side effects in vivo. **(A)** HE staining images of the heart, liver, spleen, lungs and kidneys of tumor-bearing mice after receiving different treatments. Bar: 100 μm. **(B)** Blood parameters of tumor-bearing mice after different treatments.
